# Efficient Synthesis of Novel 1,3,4-Oxadiazoles Bearing a 4-*N,N*-Dimethylaminoquinazoline Scaffold via Palladium-Catalyzed Suzuki Cross-Coupling Reactions

**DOI:** 10.3390/molecules25215150

**Published:** 2020-11-05

**Authors:** Barbara Wołek, Mateusz Werłos, Magdalena Komander, Agnieszka Kudelko

**Affiliations:** 1 Selvita Services Sp. Z o.o., Bobrzyńskiego 14, 30348 Kraków, Poland; barbara.wolek@selvita.com (B.W.); mateusz.werlos@selvita.com (M.W.); magdalena.komander@selvita.com (M.K.); 2 Department of Chemical Organic Technology and Petrochemistry, The Silesian University of Technology, Krzywoustego 4, 44100 Gliwice, Poland

**Keywords:** heterocycles, Suzuki cross-coupling, phase-transfer catalysis, palladium, catalysis

## Abstract

Two series of novel (symmetrical and unsymmetrical) quinazolinylphenyl-1,3,4-oxadiazole derivatives were synthesized using palladium-catalyzed Suzuki cross-coupling reactions. The presented synthetic methodology is based on the use of bromine-substituted 2-phenyl-4-*N,N*-dimethylaminoquinazolines and either a boronic acid pinacol ester or a diboronic acid bis(pinacol) ester of 2,5-diphenyl-1,3,4-oxadiazole. The reactions are conducted in a two-phase solvent system in the presence of catalytic amounts of [1,1′-bis(diphenylphosphino)ferrocene]-dichloropalladium(II), sodium carbonate, and tetrabutylammonium bromide, which plays the role of a phase-transfer catalyst. The luminescence properties of the obtained compounds are discussed in the context of applying these compounds in optoelectronics. Specifically, two highly-conjugated final products: *N,N*-dimethyl-2-phenyl-6-(4-(5-phenyl-1,3,4-oxadiazol-2-yl)phenyl)quinazolin-4-amine (**8f**) and 6,6′-(4,4′-(1,3,4-oxadiazole-2,5-diyl)bis(4,1-phenylene))bis(*N,N*-dimethylquinazolin-4-amine (**9f**), which contain a 1,3,4-oxadiazole moiety connected to a quinazoline ring by a 1,4-phenylene linker at the 6 position, exhibit strong fluorescence emission and high quantum yields.

## 1. Introduction

Heterocyclic compounds containing five-membered diazole or six-membered diazine scaffolds have recently attracted attention due to the fact that they participate in a wide range of biological interactions and have potential for various industrial applications. One subgroup of the diazole family includes 1,3,4-oxadiazole and its derivatives. Some of these compounds are well-known and already applied in the medicinal field, as integrase inhibitors to treat HIV (raltegravir) [1,2], hepatitis B and C virus (HBV, HCV) inhibitors [3,4], anticancer drugs (zibotentan), and as antimicrobial (furamizole) [4,5], antifungal [4,6], and antiallergic agents [7]. Because of their favorable thermal and luminescent properties, compounds based on the 1,3,4-oxadiazole ring are also candidates for optoelectronic materials, including organic light emitting diodes (OLEDs) [8,9], liquid crystals [10], metal ion sensors [11,12], and coordination polymers [13]. The most popular method for synthesizing the 1,3,4-oxadiazole scaffold involves cyclization of *N,N*′-diacylhydrazine promoted by SOCl_2_ [14], BF_3_·Et_2_O [15], TsCl and various bases including 1,8-diazabicyclo [5.4.0]undec-7-ene (DBU), Et_3_N, pyridine [16]. Alternatively, some reports have described (i) oxidative cyclization of *N*‑acylhydrazones with different oxidizing agents, such as Dess-Martin periodinane (DMP) [17], *N*‑chlorosuccinimide (NCS) and DBU [18], (diacetoxyiodo)benzene [19], KMnO_4_ [20] or 2,3-dichloro-5,6-dicyano-1,4-benzoquinone (DDQ) [21], (ii) photoisomerization of 1,2,4-oxadiazoles [22], (iii) heterocyclization of semicarbazide, thiosemicarbazide, or selenosemicarbazide derivatives [23,24], or (iv) *N*-acylation followed by ring opening/closing of tetrazoles [25,26].

Another interesting heterocyclic arrangement is quinazoline, which belongs to the subgroup of 1,3-diazines. Many representatives of this class of compound are applied in medicine as antimalarial [27], antimicrobial and anticancer agents [28,29,30], or they serve as ligands for benzodiazepine and gamma-aminobutyric acid (GABA) receptors in the central nervous system [31]. Additionally, some are applied in agrochemistry [32], and quinazoline-based nucleosides have been reported as interesting new materials exhibiting nonlinear optical or fluorescent properties, highlighting their potential use in OLEDs [33]. A wide range of synthetic procedures allow generation of the studied quinazoline derivatives, including amidation and oxidative ring closure of 2‑aminobenzoic acid derivatives (e.g., 2-aminobenzonitrile [34], 2-aminobenzoic acid [35] and 2‑aminobenzamide [36,37]), condensation of imidates with 2-aminobenzoic acid [38], or reacting anthranilate esters with guanidine [39,40,41]. An additional, recently described methodology involves cyclization of 2‑aminobenzophenones and benzylamines in the presence of *t-*BuOOH and catalytic amounts of I_2_ or ceric ammonium nitrate (CAN) [42,43].

**Figure 1:**
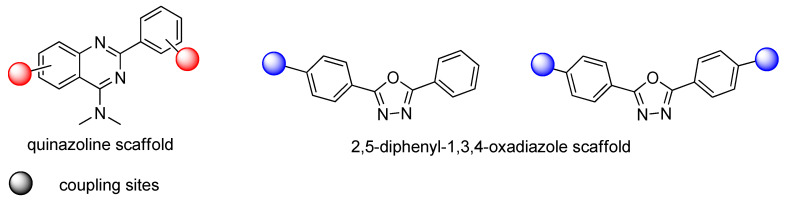
Building scaffolds of the synthesized arrangements with the indicated coupling sites.

The rapidly developing field of material sciences has directed the interest of numerous research groups towards finding new organic arrangements that exhibit interesting luminescent properties. An optimized organic luminophore is typically comprised of an extended *π*-conjugated chromophore system embodying suitable electron-hole transporting properties, high external quantum efficiency and brightness, as well as chemical and thermal stability [44,45,46]. A comprehensive literature survey revealed the possibility of modifying luminophore electron-transporting properties by direct or indirect (via the appropriate linker) conjugation of the target molecule with other electron-deficient systems, such as tetrazines, pyridines, quinolines, quinazolines, furans, thiophenes, selenophenes, or 1,3,4-oxadiazoles. [47,48,49,50,51,52,53]. Previous work conducted in our laboratory developed the synthesis of novel organic hybrids containing 1,3,4-oxadiazole, 1,3,4-thiadiazole, or 1,2,4-triazole cores conjugated via phenylene linker to different homo- and heteroaromatic arrangements, which resulted in the generation of products with high fluorescence quantum yields [53]. Building upon these results involving novel conjugated diazole-derived monomers for potential optoelectronic applications, herein we describe the combination of two different individual structural motifs (i.e., 1,3,4-oxadiazole and quinazoline) using a phenylene linker via a palladium-catalyzed Suzuki cross-coupling reaction (Figure 1). It is worth mentioning that Suzuki cross-coupling reactions play an important role in the catalytic construction of C-C bonds, and numerous papers and reviews discussing various catalytic systems, general reaction conditions, and potential applications have been published in recent years [54,55].

## 2. Results and Discussion

**Scheme 1:**
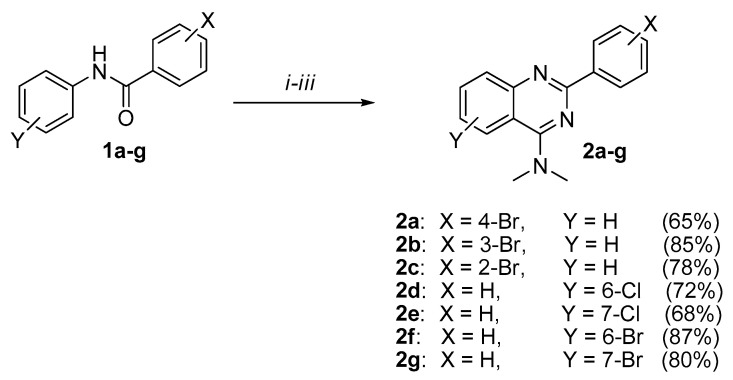
Synthesis of 4-(*N*,*N*-dimethylamino)-2-phenylquinazoline derivatives (**2a-g**). *Reagents and conditions*: (i) PCl_5_, toluene, 50 °C; (ii) Me_2_NCN, toluene, rt, 24 h; (iii) TiCl_4_, toluene, 70 °C, 5 h.

Quinazoline precursors **2a-g** were prepared in a two-step synthesis according to methodologies described in the literature [56,57,58]. The starting material was a benzamide derivative **1** substituted at the *ortho, meta*, or *para* position of the *N*-phenyl ring (X = Br, Scheme 1), or at the *meta* or *para* position of the benzamide ring (Y = Cl, Br, Scheme 1). In order to obtain the desired products **2a-g**, the benzamide derivatives **1a-g** were gently heated for few hours with PCl_5_ in anhydrous toluene, then treated with *N*,*N*-dimethylcyanamide (Me_2_NCN), and finally heated in the presence of TiCl_4_ in anhydrous toluene (Scheme 1).

**Scheme 2:**
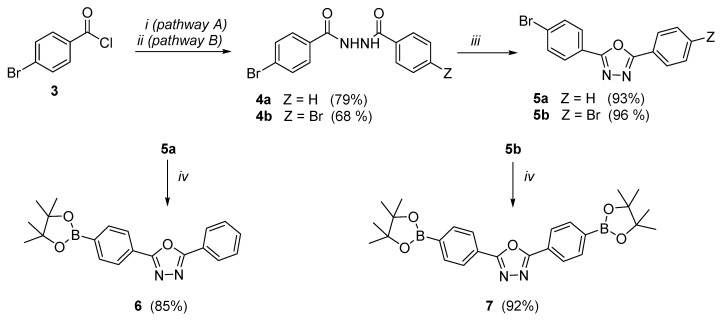
Synthesis of boronic acid pinacol ester and diboronic acid bis(pinacol) ester of 2,5‑diphenyl-1,3,4-oxadiazole (**6, 7**). *Reagents and conditions*: (i) PhCONHNH_2_, NEt_3_, CHCl_3_, 4 h, rt; (ii) N_2_H_4_·H_2_O, NEt_3_, CHCl_3_, 4 h, rt; (iii) POCl_3_, toluene, reflux, 10 h; (iv) bis(pinacolato)diboron, 1,4‑dioxane, AcOK, Pd(dppf)Cl_2_, sealed tube, 100 °C, overnight.

Meanwhile, two other reagents for Suzuki cross-coupling reactions were synthesized, specifically, the appropriate boronic acid pinacol ester and the diboronic acid bis(pinacol) ester of 2,5-diphenyl-1,3,4-oxadiazole. These transformations involved the reaction of commercially-available 4‑bromobenzoyl chloride (**3**) with either benzhydrazide (pathway A) or hydrazine hydrate (pathway B), in the presence of triethylamine. Subsequent heating of the crude *N*,*N*’-diacylhydrazines **4a,b** with phosphorus oxychloride in a non-polar solvent generated the products, 2-(4-bromophenyl)-5-phenyl-1,3,4-oxadiazole (**5a**) and 2,5-bis(4-phenyl)-1,3,4-oxadiazole (**5b**), which were then reacted with bis(pinacolato)diboron in dioxane overnight at 100 °C in the presence of potassium acetate as a base and [1,1′-bis(diphenylphosphino)ferrocene]dichloropalladium(II) Pd(dppf)Cl_2_ as a catalyst. The synthetic routes to obtaining boronic acid pinacol esters (**6**, **7**) are presented in Scheme 2.

**Scheme 3:**
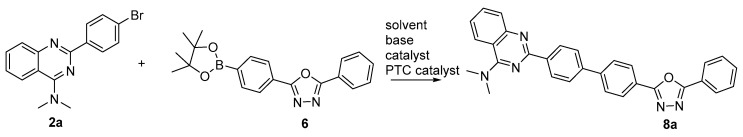
Coupling of the model quinazoline **2a** with monoboronic ester **6**.

The final step involved the Suzuki cross-coupling reaction between halogen-containing quinazolines **2a-g** and boronic derivatives, **6** and **7**. To obtain the best possible yields, the reaction conditions for the coupling reaction were optimized (Table 1). For this reason, boronic ester **6** and 4‑(*N,N*-dimethylamino)-2-phenylquinazoline substituted with bromine at the para position of the 2‑phenyl group (**2a**) were chosen as model reagents (Scheme 3).

The preliminary trials were conducted in a mixture of toluene and aqueous Na_2_CO_3_ solution in the presence of tetrakis(triphenylphosphine)palladium(0) Pd(PPh_3_)_4_, with a phase transfer catalyst (PTC), which allows regents transfer between two immiscible phases (toluene and water). Following the literature mentions regarding cross-coupling reactions assisted with phase transfer catalysts [59], three types of catalysts were tested: aliquat 336 [60], benzyltriethylammonium chloride (BnEt_3_NCl) [61], tetrabutylammonium chloride (Bu_4_NCl), or tetrabutylammonium bromide (Bu_4_NBr) [62] (Table 1, entries 1–4). 

**Scheme 4:**
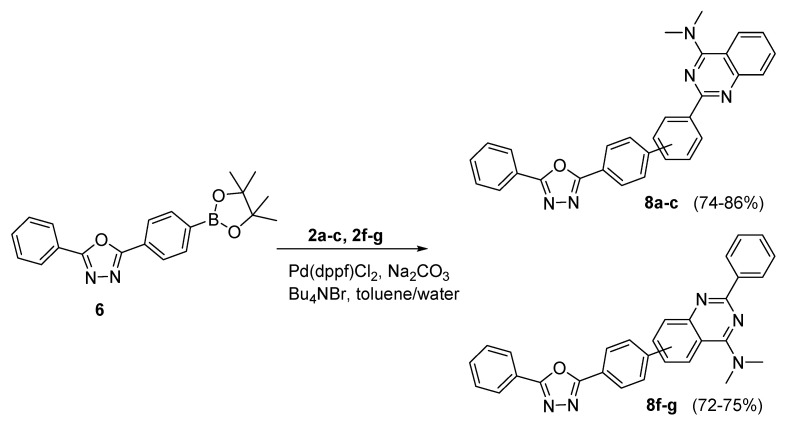
Coupling of quinazoline derivatives (**2a-c**, **2f-g**) with boronic ester **6.** *Reagents and conditions*: quinazoline derivative **2a-c, 2f-g** (0.31 mmol), boronic acid pinacol ester **6** (0.37 mmol), Pd(dppf)_2_ (0.015 mmol), Bu_4_NBr (0.03 mmol), Na_2_CO_3_ (1.53 mmol), toluene-H_2_O (3.3:1.7 mL), sealed tube, oil bath 115 °C, overnight.

All reactions led to the formation of the desired quinazolinylphenyl-1,3,4-oxadiazole derivative **8a** in similar yields (61–68%); however, the optimal result of 68% was obtained using Bu_4_NBr as the PTC (Table 1, entry 4). During the search for the optimal conditions, various palladium catalysts, such as tetrakis(triphenylphosphine)palladium(0) (Pd(PPh_3_)_4_), bis(tri-t-butylphosphine)palladium(0) (Pd(t-Bu_3_P)_2_), [1,1′-bis(diphenylphosphino)ferrocene]palladium(II) dichloride (Pd(dppf)Cl_2_), and bis(triphenylphosphine)palladium(II) dichloride (Pd(PPh_3_)_2_Cl_2_) were also tested in toluene-water media in the presence of Na_2_CO_3_ as a base (Table 1, entries 4–7). In all cases, the product, **8a**, was obtained in satisfactory yields, varying from 68 to 85%. However, the catalytic system composed of Pd(dppf)Cl_2_ and Bu_4_NBr was the most effective combination, leading to a product yield of 85% (Table 1, entry 6). Additionally, other solvents, such as dioxane, ethanol, 1,2-dimethoxyethane (DME), and dimethylformamide (DMF) (Table 1, entries 11–14) were tested, but overall, the best results were achieved using a two-phase toluene-water mixture (Table 1, entry 6). Optimization of the synthetic conditions also required investigating the influence of the type of base used in the reaction, which participates in (i) halogen ion exchange involving the initial halide-containing reagent, **2a**, at the surface of the catalyst, and (ii) activation of boronic ester, **6**, to facilitate transmetalation. Among the different bases tested, including Na_2_CO_3_, K_2_CO_3_, t‑BuOK, and AcONa, the best results were obtained after using Na_2_CO_3_ (85% yield; Table 1, entry 6). Therefore, these optimized reaction conditions were applied in the synthesis of unsymmetrical quinazolinylphenyl-1,3,4-oxadiazole derivatives (**8a-c, 8f‑g**). The key boronic ester (**6**) reacted with various halogen-containing 4-(*N,N*-dimethylamino)-2-phenylquinazoline derivatives (**2a-g**) in the presence of 5 mol% palladium catalyst (Pd(dppf)Cl_2_), Na_2_CO_3_, and Bu_4_NBr as a PTC catalyst, in the two-phase solvent system (toluene-H_2_O). The mixtures were heated overnight in a sealed glass reactor, and the products were purified by extraction, followed by column chromatography and trituration. The results of the cross-coupling reactions are presented in Table 2. This study afforded several novel unsymmetrical quinazolinylphenyl-1,3,4-oxadiazole derivatives (**8a-c, 8f-g**) in satisfactory yields (72–86%; Scheme 4).

Syntheses of quinazoline regioisomers **2a-2c**, substituted with bromine at the 2-phenyl ring position, were successful, and the products, **8a-c**, were obtained in high yields (74–86%; Table 2, entries 1‑3). However, the yield of product, **8b**, which was derived from the meta-substituted arrangement (**2b**) was slightly lower (74%; Table 2, entry 2) than for products obtained from para- and ortho-substituted derivatives (82–86%; Table 2, entries 1,3). These results can be explained based on the electron-withdrawing nature of the quinazoline scaffold adjacent to the phenyl ring. The same conditions were applied for 4-(*N*,*N*-dimethylamino)-2-phenylquinazolines **2d**, **2e** substituted at positions 6 and 7 with chlorine atoms, where no conversion was observed (Table 2, entries 4,5). For the same substrates **2d**, **2e**, additional reactions in the microwave reactor were performed, but no positive outcomes were obtained. In contrast, the same coupling reactions conducted for the 6- and 7-bromide variations resulted in the formation of the desired products **8f-g** in satisfactory yields (72–75%; Table 2). This observation confirms the general trend in the reactivity of aryl halides in Suzuki cross-coupling reactions, where bromine derivatives are much more reactive than their chloride analogues.

**Scheme 5:**
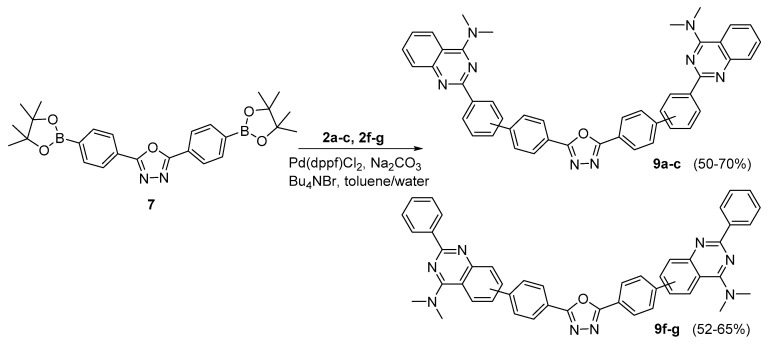
Coupling of quinazoline derivatives (**2a-c**, **2f-g**) with boronic ester **7**. *Reagents and conditions*: quinazoline derivative **2a-c, 2f-g** (0.50 mmol), diboronic acid bis(pinacol) ester **7** (0.28 mmol), Pd(dppf)Cl_2_ (0.021 mmol), Bu_4_NBr (0.042 mmol), Na_2_CO_3_ (2.11 mmol), toluene-H_2_O (6.6:3.4 mL), sealed tube, oil bath 115 °C, overnight.

Applying the described optimized conditions, another series of reactions was carried out with symmetrical diboronic acid bis(pinacol)ester, **7**, and 4-(*N*,*N*-dimethylamine)-2-phenylquinazoline derivatives **2a-c**, **2f-g** in the presence of 10 mol% palladium catalyst (Pd(dppf)Cl_2_), Na_2_CO_3_, and tetrabutylammonium bromide in the two-phase toluene-water solvent system (Scheme 5). The mixture was heated overnight in a sealed glass reactor, and the products were purified by extraction, followed by column chromatography and trituration.

The results are presented in Table 3. This study afforded novel, symmetrical 2,5-bis(quinazolinylphenyl)-1,3,4-oxadiazole derivatives **9a-c**, **9f-g** in satisfactory yields (50–70%; Table 3). The best results were obtained for **9a**, where the diboronic precursor containing a 2,5-diphenyl-1,3,4-oxadiazole scaffold **7** was coupled to quinazoline arrangement, **2a**, with a 1,4-phenylene linker (70%; Table 3, entry 2). In general, the derivatives, **9a-c**, exhibited a similar trend in yields, as was observed for the analogous mono-substituted entities **8a‑c**. Specifically, the meta derivative was produced in lower yield relative to the ortho- and para-substituted counterparts. It was also determined that the symmetrically substituted derivatives **9a‑c**, **9f-g** were obtained in lower yields compared with the unsymmetrical arrangements **8a-c**, **8f-g**, because of their relatively lower solubility in most organic solvents, which complicated the purification procedure.

The structures of unsymmetrical and symmetrical quinazolinylphenyl-1,3,4-oxadiazole derivatives **8a-c**, **8f-g**, **9a-c**, **9f-g** obtained following Suzuki cross-coupling reactions involving the boronic precursors **6**, **7** containing a 1,3,4-oxadiazole scaffold were confirmed using typical spectroscopic methods (nuclear magnetic resonance spectroscopy ^1^H- and ^13^C-NMR, UV-Vis spectrometry, high resolution mass spectrometry HRMS, infrared spectroscopy IR, see Supplementary Materials).

In the ^1^H-NMR spectra of unsymmetrical (compounds **8a-c**, **8f-g**) and symmetrical quinazolinylphenyl-1,3,4-oxadiazole derivatives (compounds **9a-c**, **9f-g**), characteristic signals from the dimethylamino group are observed in the range between 2.94–3.55 ppm.

Two protons from the phenyl group for **8a-b**, **8f-g** and **9a-b**, **9f-g**, substituted at the quinazoline moiety, are shifted to lower field, and appear in the range between 8.50–8.90 ppm. In the remaining derivatives, **8c** and **9c**, these protons are observed in the range of 8.15–8.18 ppm. Such significant changes in chemical shifts are likely due to the proximity of these protons to the electronegative nitrogen atoms. In the ^13^C-NMR spectra, the characteristic signals from the dimethylamino group are observed in the narrow range between 41.4–42.1 ppm. The unsymmetrical quinazolinylphenyl-1,3,4-oxadiazole derivatives (**8a-c**, **8f-g**), have four distinctive signals from the two quaternary quinazoline carbons C-4a and C-8a, as well as carbon atoms C-2 and C-5 of the 1,3,4-oxadiazole ring, which appear in the range of 158.7–164.7 ppm. However, in the symmetrical quinazolinylphenyl-1,3,4-oxadiazole derivatives (**9a-c**, **9f-g**) the signals range from 158.9 to 164.7 ppm. Both the ^1^H- and ^13^C-NMR spectra of 2,5-bis(4-arylphenyl)-1,3,4-oxadiazoles (**9a-c**, **9f‑g**) display a reduced number of peaks due to the symmetrical nature of these compounds’ structures.

UV-Vis spectra of the investigated compounds **8a-c**, **8f-g**, **9a-c**, **9f-g**, measured in dichloromethane (DCM), or methanol (MeOH) revealed the presence of three, four or five absorption maxima, depending on the symmetry and structure of the connection between the 1,3,4-oxadiazole moiety and the 4-*N,N*-dimethylaminoquinazoline scaffold. Longwave absorption bands (n→π*) of the symmetrical **9a-c**, **9f-g** and unsymmetrical **8a-c**, **8f-g** quinazoline derivatives were located in the range of 302–361 nm and characterized by high absorption coefficients, comparable to π→π* absorption bands. The fluorescence spectra of the studied compounds **8a-c**, **8f-g**, **9a-c**, **9f-g** are generally complex and composed of multiple individual signals with considerable Stokes shifts (∆), varying from 58 to 131 nm (Table 4).

The quantum yields (Φ_f_) were determined based on two standards (i.e., 1,4-diphenylbuta-1,3-diene (DPB) and 9,10-diphenylanthracene (DPA) in non-polar solvents [63,64], according to the method described by Brouwer [65]. The analysis of Stokes shifts showed that the quinazolinylphenyl-1,3,4-oxadiazoles **8a-c**, **8f-g**, representing the series of unsymmetrical arrangements composed of six conjugated and fused aromatic rings, exhibited relatively higher values of Stokes shifts (60–131 nm, Table 4, entries 3–7) in contrast to the corresponding symmetrical derivatives **9a-c**, **9f-g**, containing the more extended arrangement of nine aromatic rings (58–108 nm, Table 4, entries 8–12). Moreover, the introduction of the additional aromatic rings of 4-*N*,*N*-dimethylamino-2-phenylquinazoline scaffold, and the lenghtening of the conjugation in **9a-c**, **9f-g** series did not lead to both a significant increase in Stokes shifts and quantum yields, except for the symmetrical derivative **9f** (Table 4, entry 11). Generally, the highest quantum yields were achieved for the unsymmetrical **8f** and symmetrical **9f** compounds (0.61 and 0.98 respectively, Table 4, entries 6, 11), in which the 1,3,4-oxadiazole moiety is connected to the quinazoline by a 1,4-phenylene linker at the 6 position, which allowed effective electron transfer between these two heterocyclic moieties. Noteworthy is the fact that the irradiation of **8f** by UV radiation led to violet fluorescence that was visible to the naked eye. It is important to note that the boronic acid pinacol ester and diboronic acid bis(pinacol) ester of 2,5-diphenyl-1,3,4-oxadiazole (**6**, **7**) starting compounds, which play a role in building the scaffolds for the studied conjugated arrangements, also had high quantum yields (0.68 for **6**, and 0.91 for **7**; Table 4, entries 1,2). However, the other products formed from the conjugation of boronic esters (**6**, **7**) to quinazolines containing a *N*,*N*-dimethylamino group did not exhibit increased quantum yields. In fact, they caused fluorescence quenching, such that their quantum yields did not exceed 0.13 (**8a-c**, **8g**, **9a-c**, **9g**; Table 4, entries 3–5,7,8–10,12). A survey of the literature revealed that electron-rich species, including substituted amino groups that can donate electrons to aromatic heterocycles (i.e., fluorophores), can act as potential fluorescence quenchers [66]. They are responsible for the formation of charge transfer complexes, which often return to the ground state without emitting a photon.

## 3. Materials and Methods

### 3.1. General Information

All solvents and reagents were purchased from commercial sources and were used without additional purification. Melting points (mp) were measured using a SMP3 melting point apparatus (Stuart, Staffordshire, UK) and are uncorrected. UV-Vis spectra were recorded on a Jasco V-650 (Jasco Corporation, Tokyo, Japan) or a U-3900H spectrophotometer (Hitachi, Tokyo, Japan). Elemental analysis were performed with a VarioEL analyser (Elementar UK Ltd., Stockport, UK). ^1^H- (400 MHz) and ^13^C-NMR spectra (101 MHz) were recorded in CDCl_3_ solutions using TMS as internal standard on an Agilent 400-NMR spectrometer (Agilent Technologies, Waldbronn, Germany). Thin layer chromatography (TLC) was performed on silica gel 60 F_254_ TLC plates (Merck KGaA, Darmstadt, Germany) using benzene-EtOAc (3:1), DCM-EtOAc (9:1), DCM-MeOH (95:5) as the mobile phases. FT-IR spectra were recorded between 4000 and 6500 cm^−1^ on a Nicolet 6700 FT-IR apparatus (Thermo Fischer Scientific, Wesel, Germany) equipped with a Smart iTR accessory. High-resolution mass spectra (HRMS) were acquired on an Ultra-High Resolution (UHR) mass spectrometer Impact II^TM^ QTOF instrument (Bruker, Bremen, Germany) equipped with an electrospray ionization (ESI) source, using MeOH or DCM as a solvent. Fluorescence spectra were recorded in DCM solution using a Hitachi F-7000 fluorescence spectrophotometer (Hitachi Ltd., Tokyo, Japan) at room temperature.

### 3.2. Synthesis and Characterization

#### 3.2.1. General Procedure for Preparing 4-(*N*,*N*-dimethylamino)-2-phenylquinazoline Derivatives 2a-g

The appropriate benzamide derivative (0.025 mol): *N*-phenyl-4-bromobenzamide (**1a**), *N*‑phenyl-3-bromobenzamide (**1b**), *N*-phenyl-2-bromobenzamide (**1c**), *N*-(4-chlorophenyl)-benzamide (**1d**), *N*-(3-chlorophenyl)benzamide (**1e**), *N*-(4-bromophenyl)benzamide (**1f**) or *N*-(3-bromophenyl)benzamide (**1g**) and PCl_5_ (5.75 g; 0.028 mol) were gently heated in anhydrous toluene (50 mL) at about 50 °C until the benzamide derivative was completely consumed (based on TLC, 3–6 h). The mixture was concentrated on a rotary evaporator to remove toluene and POCl_3_. Anhydrous toluene (50 mL) and *N,N*-dimethylcyanamide (1.75 g; 2.0 mL; 0.025 mol) were added, and the mixture was left for 24 h at room temperature before dropwise addition of a solution of TiCl_4_ (2.5 mL; 0.025 mol) in 10 mL of anhydrous toluene. The mixture was agitated at elevated temperature (70 °C) for 5 h. After cooling, the solvent was decanted from the gluey solid, and 100 mL of 20% aqueous HCl added. The hydrolyzed mixture was filtered, and the resulting solution was neutralized with 20% aqueous NaOH. The precipitate was extracted with chloroform (3 × 20 mL), dried and concentrated. The crude products were purified by column chromatography (silica gel, eluent: benzene-EtOAc, 3:1) to yield the appropriate 4-(*N,N*-dimethylamino)-2-phenylquinazoline derivatives **2a-g**.

*2-(4-Bromophenyl)-4-(N,N-dimethylamino)quinazoline* (**2a**). The product was obtained as a colourless solid (5.33 g, 65%); mp 104–105 °C (108–110 °C [67]); *R_f_* = 0.54 (benzene-EtOAc, 3:1).

*2-(3-Bromophenyl)-4-(N,N-dimethylamino)quinazoline* (**2b**). The product was obtained as a colourless solid (6.97 g, 85%); mp 82–84 °C; *R_f_* = 0.52 (benzene-EtOAc, 3:1). ^1^H-NMR (CDCl_3_): δ = 3.42 (s, 6H); 7.33 (t, *J* = 7.6 Hz, 1H); 7.37 (t, *J* = 8.4 Hz, 1H); 7.56 (d, *J* = 7.6 Hz, 1H,); 7.69 (t, *J* = 8.8 Hz, 1H); 7.91 (d, *J* = 8.4 Hz, 1H); 8.00 (d, *J* = 8.8 Hz, 1H); 8.49 (d, *J* = 7.6 Hz, 1H); 8.70 (s, 1H). ^13^C- NMR (CDCl_3_): δ = 41.8; 115.0; 122.5; 124.3; 125.5; 126.9; 128.7; 129.7; 131.3; 132.0; 141.0; 152.8; 157.8; 163.8. Anal. Calcd for C_16_H_14_N_3_Br: C, 58.55; H, 4.30; N, 12.80. Found: C, 58.49; H, 4.26; N, 12.82.

*2-(2-Bromophenyl)-4-(N,N-dimethylamino)quinazoline* (**2c**). The product was obtained as a colourless solid (6.40 g, 78%); mp 148–150 °C; *R_f_* = 0.56 (benzene-EtOAc, 3:1). ^1^H-NMR (CDCl_3_): δ = 3.41 (s, 6H); 7.24 (t, *J* = 7.6 Hz, 1H); 7.39 (t, *J* = 8.8 Hz, 1H); 7.41 (t, *J* = 7.6 Hz, 1H); 7.67 (d, *J* = 7.6 Hz, 1H); 7.72 (t, *J* = 7.2 Hz, 1H); 7.82 (d, *J* = 7.2 Hz, 1H); 7.94 (d, *J* = 8.8 Hz, 1H); 8.05 (d, *J* = 7.2 Hz, 1H). ^13^C-NMR (CDCl_3_): δ = 41.9; 114.5; 121.8; 124.5; 125.4; 127.2; 128.6; 129.7; 131.5; 132.0; 133.6; 140.9; 152.4; 161.1; 163.3. Anal. Calcd for C_16_H_14_N_3_Br: C, 58.55; H, 4.30; N, 12.80. Found: C, 58.50; H, 4.28; N, 12.84.

*6-Chloro-4-(N,N-dimethylamino)-2-phenylquinazoline* (**2d**). The product was obtained as a colourless solid (5.10 g, 72%); mp 95–97 °C (94–96 °C [68]); *R_f_* = 0.62 (benzene-EtOAc, 3:1).

*7-Chloro-4-(N,N-dimethylamino)-2-phenylquinazoline* (**2e**). The product was obtained as a beige solid (4.82 g, 68%); mp 138–139 °C; *R_f_* = 0.59 (benzene-EtOAc, 3:1). ^1^H-NMR (CDCl_3_): δ = 3.39 (s, 6H); 7.24 (d, *J* = 8.8 Hz, 1H); 7.46–7.48 (m, 3H); 7.80–7.90 (m, 2H); 8.52 (d, *J* = 8.0 Hz, 2H). ^13^C-NMR (CDCl_3_): δ = 41.7; 113.1; 124.6; 126.8; 127.6; 128.2; 128.4; 130.2; 137.8; 138.5; 153.9; 160.1; 163.3. Anal. Calcd for C_16_H_14_N_3_Cl: C, 67.72; H, 4.97; N, 14.81. Found: C, 67.68; H, 4.95; N, 14.76.

*6-Bromo-4-(N,N-dimethylamino)-2-phenylquinazoline* (**2f**). The product was obtained as a yellowish solid (7.14 g, 87%); mp 101–102 °C (99–100 °C [68]); *R_f_* = 0.56 (benzene-EtOAc, 3:1).

*7-Bromo-4-(N,N-dimethylamino)-2-phenylquinazoline* (**2g**). The product was obtained as a colourless solid (6.56 g, 80%); mp 135–136 °C; *R_f_* = 0.50 (benzene-EtOAc, 3:1). ^1^H-NMR (CDCl_3_): δ = 3.39 (s, 6H); 7.39 (d, *J* = 8.8 Hz, 1H); 7.44–7.49 (m, 3H); 7.81 (d, *J* = 8.8 Hz, 1H); 8.10 (s, 1H); 8.51–8.54 (m, 2H). ^13^C-NMR (CDCl_3_): δ = 41.7; 113.4; 126.3; 126.8; 127.1; 128.2; 128.4; 130.3; 130.9; 138.4; 153.9; 159.9; 163.4. Anal. Calcd for C_16_H_14_N_3_Br: C, 58.55; H, 4.30; N, 12.80. Found: C, 58.57; H, 4.33; N, 12.85.

#### 3.2.2. Preparation of 2-(4-Bromophenyl)-5-phenyl-1,3,4-oxadiazole (**5a**)—Pathway A

4-Bromobenzoyl chloride (**3**, 10.98 g, 0.05 mol) was added to a magnetically agitated solution of benzhydrazide (6.81 g, 0.05 mol) and triethylamine (7.0 mL, 0.05 mol) in 100 mL of chloroform, which was placed in an ice bath, and once the addition was complete, the mixture was stirred for 4 h at room temperature. The solid precipitate was collected by filtration, washed with hexane (50 mL) and a large quantity of water (200 mL), air-dried, yielding 12.60 g (79% yield) of pure *N’*-benzoyl-4-bromobenzohydrazide (**4a**). The crude intermediate, **4a**, was treated with phosphorous oxychloride (25 mL, 0.27 mol) and added to 100 mL of dry toluene, and the mixture was refluxed until the initial **4a** was fully consumed (based on TLC, 10 h). After cooling, it was concentrated, and the precipitated crystals were filtered off to give pure 2-(4-bromophenyl)-5-phenyl-1,3,4-oxadiazole (**5a**). Colourless crystals (11.05 g, 93%); mp 169–170 °C (169–170 °C [69]); R*_f_* = 0.60 (benzene-EtOAc, 3:1).

#### 3.2.3. Preparation of 2,5-bis(4-Bromophenyl)-1,3,4-oxadiazole (**5b**)—Pathway B

4-Bromobenzoyl chloride (**3**, 21.95 g, 0.10 mol) was added to a magnetically agitated solution of hydrazine hydrate (2.4 mL, 0.05 mol) and triethylamine (13.9 mL, 0.10 mol) in 100 mL of chloroform, which was placed in an ice bath. After the addition, the mixture was stirred for 4 h at room temperature. The solid precipitate was collected by filtration, washed with hexane (50 mL) and a large quantity of water (200 mL), and air-dried, yielding 13.53 g (68% yield) of pure *N,N’*-bis(4-bromobenzoyl)hydrazine (**4b**). The crude intermediate, **4b**, was treated with phosphorous oxychloride (25 mL, 0.27 mol) and added to 100 mL of dry toluene, and the mixture was refluxed until the starting material, **4b**, was fully consumed (based on TLC, 10 h). After cooling, it was concentrated, and the precipitated crystals were filtered off to give pure 2,5-bis(4-bromophenyl)-1,3,4-oxadiazole (**5b**). Colourless crystals (12.40 g, 96%); mp 259 °C (258 °C [70]); R*f* = 0.62 (benzene-EtOAc, 3:1).

#### 3.2.4. Preparation of 2-Phenyl-5-[4-(tetramethyl-1,3,2-dioxaborolan-2-yl)phenyl]-1,3,4-oxadiazole (**6**)

Bis(pinacolato)diboron (2.42 g; 0.0095 mol) and AcOK (1.95 g; 0.020 mol) were added to a degassed 1,4-dioxane (15 mL) solution of 2,5-bis(4-bromophenyl)-1,3,4-oxadiazole (**5b**) (1.50 g; 0.004 mol) in a glass tube reactor. The mixture was bubbled with Ar for 15 min, and then Pd(dppf)Cl_2_ (0.15 g; 0.0002 mol) was added. The glass tube reactor was sealed, and the reaction was stirred at 100 °C overnight. The solvent was evaporated, and the residue was portioned between DCM and H_2_O. The resulting mixture was extracted with DCM twice (2 × 30 mL). The final product was purified by column chromatography (silica gel, eluent: 0–10% EtOAc in DCM), followed by trituration with *n*‑hexane to obtain 1.48 g of pure product **6**. Cream solid (1.48 g, 85%); mp 167 °C; R*_f_* = 0.59 (DCM-EtOAc, 9:1). IR (ATR): 2975, 1614, 1571, 1541, 1485, 1393, 1407, 1354, 1331, 1269, 1211, 1141, 1114, 1089, 1069, 1026, 1016, 964, 890, 847, 821, 776, 717, 700, 690, 667 cm^−1^. ^1^H-NMR (CDCl_3_): δ = 8.23–8.06 (m, 4H), 8.03–7.92 (m, 2H), 7.61–7.49 (m, 3H), 1.38 (s, 12H). ^13^C-NMR (CDCl_3_) δ = 164.8, 164.8, 135.5, 131.9, 129.2, 127.1, 126.2, 126.1, 124.1, 84.4, 67.2, 25.0. HRMS (ESI/Q-TOF) *m/z*: [M + H]^+^ calcd for C_20_H_22_BN_2_O_3_ 349.1718; found 349.1755. UV (MeOH): λ_max_ (ε) = 203.5 (26630), 237.5 (7930), 287.5 (32610).

#### 3.2.5. Preparation of bis[4-(Tetramethyl-1,3,2-dioxaborolan-2-yl)phenyl]-1,3,4-oxadiazole (**7**)

Bis(pinacolato)diboron (2.42 g; 0.0095 mol) and AcOK (1.95 g; 0.020 mol) were added to a degassed 1,4-dioxane (15 mL) solution of 2,5-bis(4-bromophenyl)-1,3,4-oxadiazole (**5b**) (1.50 g; 0.004 mol) in a glass tube reactor. The mixture was bubbled with Ar for 15 min, and then Pd(dppf)Cl_2_ (0.15 g; 0.0002 mol) was added. The glass tube reactor was sealed, and the reaction was stirred at 100 °C overnight. The solvent was evaporated, and the residue was portioned between DCM and H_2_O. The resulting mixture was extracted with DCM twice (2 × 30 mL). The final product was purified by column chromatography (silica gel, eluent: 0–10% EtOAc in DCM), followed by trituration with *n‑*hexane to obtain 1.73 g of pure product **7**. Pink pale solid (1.73 g, 92%); mp 248 °C (decomp.) (249 °C [24]); R*_f_* = 0.51 (DCM-EtOAc, 9:1). IR (ATR): 2980, 1615, 1568, 1543, 1411, 1396, 1356, 1327, 1268, 1210, 1167, 1141, 1092, 1016, 964, 88, 847, 821, 715, 667 cm^−1^. ^1^H-NMR (300 MHz, CDCl_3_): δ = 8.15 (d, *J* = 8.4 Hz, 4H), 7.96 (d, *J* = 8.4 Hz, 4H), 1.38 (s, 24H). ^13^C-NMR (75 MHz, CDCl_3_): δ = 164.9, 135.5, 132.8, 126.2, 122.8, 84.4, 25.0. HRMS (ESI/Q-TOF) *m/z*: [M + H]^+^ calcd for C_26_H_33_B_2_N_2_O_5_ 475.2576; found 475.2574. UV (MeOH): λ_max_ (ε) = 207.5 (39430), 243.0 (13340), 291.5 (37080).

#### 3.2.6. General Procedure for the Preparation of Unsymmetrical Quinazolinylphenyl-1,3,4-Oxadiazole Derivatives **8a-c**, **8f**,**g** via Suzuki Cross-Coupling from Boronic Acid Pinacol Ester **6**

The appropriate halogen-containing quinazoline derivative (0.31 mmol): 2-(4-bromophenyl)-4-(*N,N*-dimethylamino)quinazoline (**2a**), 2-(3-bromophenyl)-4-(*N,N*-dimethylamino)quinazoline (**2b**), 2-(2-bromophenyl)-4-(*N,N*-dimethylamino)quinazoline (**2c**), 6-chloro-4-(*N,N*-dimethylamino)-2-phenylquinazoline (**2d**), 7-chloro-4-(*N,N*-dimethylamino)-2-phenylquinazoline (**2e**), 6-bromo-4-(*N,N*-dimethylamino)-2-phenylquinazoline (**2f**), 7-bromo-4-(*N,N*-dimethylamino)-2-phenyl-quinazoline (**2g**), 2-phenyl-5-[4-(tetramethyl-1,3,2-dioxaborolan-2-yl)phenyl]-1,3,4-oxadiazole (**6**) (128 mg; 0.37 mmol), Na_2_CO_3_ (162 mg; 1.53 mmol), Bu_4_NBr (10 mg; 0.03 mmol) were dissolved in toluene (3.3 mL) and H_2_O (1.7 mL) in glass tube reactor, and the mixture was bubbled with Ar followed by addition of Pd(dppf)Cl_2_ (11 mg; 0.015 mmol). Glass tube reactor was sealed, and the reaction was stirred at 115 °C overnight. The solvent was evaporated, and the residue was portioned between DCM and H_2_O. The resulting mixture was extracted with DCM twice (2 × 10 mL). Product was purified by column chromatography (silica gel, eluent: 0–10% EtOAc in DCM) followed by trituration with *n-*hexane or EtOAc to yield the appropriate derivatives **8a-c, 8f-g**. In the reactions of **2d** and **2e**, no conversion was observed.

*N,N-Dimethyl-2-(4′-(5-phenyl-1,3,4-oxadiazol-2-yl)biphenyl-4-yl)quinazolin-4-amine* (**8a**). The product was obtained as a beige solid (122 mg, 85%); mp 216 °C; R*_f_* = 0.15 (DCM-EtOAc, 9:1). IR (ATR): 2939, 1610, 1562, 1551, 1523, 1484, 1449, 1422, 1400, 1375, 1335, 1266, 1215, 1177, 1125, 1105, 1070, 1028, 1005, 973, 954, 921, 878, 870, 846, 830, 799, 766, 734, 709, 701, 682 cm^−1^. ^1^H-NMR (300 MHz, CDCl_3_) δ = 8.70 (d, *J* = 8.2 Hz, 2H), 8.25 (d, *J* = 8.2 Hz, 2H), 8.22–8.16 (m, 2H), 8.03 (dd, *J* = 23.5, 8.2 Hz, 2H), 7.84 (dd, *J* = 20.0, 8.2 Hz, 4H), 7.74 (t, *J* = 7.8 Hz, 1H), 7.62–7.53 (m, 3H), 7.41 (t, *J* = 7. Hz, 1H), 3.49 (s, 6H). ^13^C-NMR (75 MHz, CDCl_3_) δ = 164.6, 164.5, 163.9, 158.7, 153.0, 144.2, 141.1, 138.8, 132.1, 131.7, 129.1, 129.0, 128.7, 127.7, 127.4, 127.0, 127.0, 125.6, 124.2, 124.0, 122.8, 115.0, 41.9. HRMS (ESI/Q-TOF) *m/z:* [M + H]^+^ calcd for C_30_H_24_N_5_O 470.1981; found 470.1983. UV (MeOH): λ_max_ (ε) = 201.5 (44520), 214.0 (19760), 316.5 (50600).

*N,N-Dimethyl-2-(4′-(5-phenyl-1,3,4-oxadiazol-2-yl)biphenyl-3-yl)quinazolin-4-amine* (**8b**). The product was obtained as a beige solid (106 mg, 74%); mp 177 °C; R*_f_* = 0.21 (DCM-EtOAc, 9:1). IR (ATR): 3060, 2880, 1612, 1564, 1526, 1502, 1485, 1476, 1456, 1413, 1395, 1375, 1359, 1332, 1270, 1231, 1189, 1163, 1100, 1072, 1042, 1026, 1016, 959, 920, 848, 819, 799, 788, 773, 727, 712, 749, 697, 686 cm^−1^. ^1^H-NMR (300 MHz, CDCl_3_) δ = 8.90 (t, *J* = 1.8 Hz, 1H), 8.63 (dt, *J* = 7.8, 1.4 Hz, 1H), 8.30–8.22 (m, 2H), 8.22–8.14 (m, 2H), 8.06 (dd, *J* = 8.3, 1.4 Hz, 1H), 7.99 (dd, *J* = 8.5, 1.4 Hz, 1H), 7.95–7.88 (m, 2H), 7.80−7.67 (m, 2H), 7.66–7.50 (m, 4H), 7.40 (ddd, *J* = 8.3, 6.9, 1.3 Hz, 1H), 3.48 (s, 6H). ^13^C-NMR (75 MHz, CDCl_3_) δ = 164.6, 164.6, 163.9, 158.9, 153.0, 144.7, 139.7, 139.7, 132.1, 131.7, 129.1, 128.9, 128.8, 128.6, 128.3, 127.8, 127.4, 127.1, 127.0, 125.6, 124.2, 124.0, 122.6, 115.0, 41.8. HRMS (ESI/Q-TOF) *m/z*: [M + H]^+^ calcd for C_30_H_24_N_5_O 470.1981; found 470.2014. UV (MeOH): λ_max_ (ε) = 201.5 (75350), 217.5 (52110), 304.0 (66480).

*N,N-Dimethyl-2-(4′-(5-phenyl-1,3,4-oxadiazol-2-yl)biphenyl-2-yl)quinazolin-4-amine* (**8c**). The product was obtained as a beige solid (123 mg, 86%); mp 172 °C; R*_f_* = 0.10 (DCM-EtOAc, 9:1). IR (ATR): 3053, 2029, 1613, 1560, 1548, 1520, 1481, 1444, 1411, 1401, 1381, 1345, 1329, 1269, 1219, 1180, 1095, 1075, 1050, 1022, 1006, 955, 920, 838, 760, 740, 734, 707, 682 cm^−1^. ^1^H-NMR (300 MHz, CDCl_3_) δ = 8.18–8.10 (m, 3H), 8.05−7.99 (m, 2H), 7.96 (dd, *J* = 8.5, 1.3 Hz, 1H), 7.91 (dd, *J* = 8.5, 1.3 Hz, 1H), 7.71 (ddd, *J* = 8.5, 6.9, 1.4 Hz, 1H), 7.60–7.48 (m, 6H), 7.45–7.38 (m, 3H), 2.95 (s, 6H). ^13^C-NMR (75 MHz, CDCl_3_) δ = 164.7, 164.5, 162.8, 161.8, 152.7, 147.0, 140.5, 139.3, 132.1, 131.7, 131.0, 130.5, 129.9, 129.1, 129.1, 128.6, 128.2, 126.9, 126.3, 125.5, 124.4, 124.0, 121.5, 114.3, 41.4. HRMS (ESI/Q-TOF) *m/z*: [M + H]^+^ calcd for C_30_H_24_N_5_O 470.1981; found 470.1983. UV (MeOH): λ_max_ (ε) = 201.5 (59880), 211.0 (49960), 243.5 (30930), 302.5 (43720).

*N,N-Dimethyl-2-phenyl-6-(4-(5-phenyl-1,3,4-oxadiazol-2-yl)phenyl)quinazolin-4-amine* (**8f**). The product was obtained as a yellowish solid (107 mg, 75%); mp 234 °C; R*_f_* = 0.24 (DCM-EtOAc, 9:1). IR (ATR): 3067, 2890, 2169, 1612, 1579, 15512, 1523, 1505, 1486, 1449, 1417, 1396, 1352, 1325, 1243, 1172, 1131, 1104, 1069, 1027, 1015, 959, 891, 852, 829, 803, 790, 776, 743, 733, 713, 692, 684, 673 cm^−1^. ^1^H-NMR (300 MHz, CDCl_3_) δ = 8.61 (dd, *J* = 7.7, 2.0 Hz, 2H), 8.32–8.23 (m, 3H), 8.23–8.15 (m, 2H), 8.11–7.97 (m, 2H), 7.88–7.78 (m, 2H), 7.63–7.46 (m, 6H), 3.53 (s, 6H). ^13^C-NMR (75 MHz, CDCl_3_) δ = 164.7, 164.4, 164.1, 159.7, 152.9, 143.8, 138.7, 135.4, 131.8, 131.0, 130.2, 129.5, 129.1, 128.4, 128.3, 127.6, 127.6, 127.0, 124.0, 123.9, 122.9, 115.2, 41.9. HRMS (ESI/Q-TOF) *m/z*: [M + H]^+^ calcd for C_30_H_24_N_5_O 470.1981; found 470.1986. UV (DCM): λ_max_ (ε) = 228.5 (36300), 297.0 (56350), 354.5 (44090).

*N,N-Dimethyl-2-phenyl-7-(4-(5-phenyl-1,3,4-oxadiazol-2-yl)phenyl)quinazolin-4-amine* (**8g**). The product was obtained as a colourless solid (104 mg, 72%); mp 238–239 °C; R*_f_* = 0.27 (DCM-EtOAc, 9:1). IR (ATR): 3061, 2862, 1615, 1560, 1533, 1480, 1449, 1428, 1416, 1398, 1381, 1348, 1269, 1235, 1197, 1158, 1141, 1125, 1077, 1064, 1031, 1018, 992, 956, 911, 869, 843, 823, 797, 778, 760, 740, 732, 703 cm^−1^. ^1^H-NMR (300 MHz, CDCl_3_) δ = 8.66–8.56 (m, 2H), 8.31–8.20 (m, 3H), 8.23–8.08 (m, 3H), 7.93 (d, *J* = 8.0 Hz, 2H), 7.66 (dd, *J* = 8.7, 2.0 Hz, 1H), 7.62–7.47 (m, 6H), 3.49 (s, 6H). ^13^C-NMR (75 MHz, CDCl_3_) δ = 164.7, 164.4, 163.5, 159.9, 153.5, 143.0, 142.9, 138.8, 131.8, 130.1, 129.1, 128.4, 128.3, 127.9, 127.5, 127.0, 126.6, 126.3, 123.9, 123.5, 122.9, 114.4, 41.8. HRMS (ESI/Q-TOF) *m/z*: [M + H]^+^ calcd for C_30_H_24_N_5_O 470.1981; found 470.1993. UV (DCM): λ_max_ (ε) = 226.5 (33990), 267.5 (42800), 314.0 (74470).

#### 3.2.7. General Procedure for Preparing Symmetrical Quinazolinylphenyl-1,3,4-oxadiazole Derivatives **9a-c**, **9f**,**g** via Suzuki Cross-Coupling Using Diboronic Acid bis(pinacol) Ester **7**

The appropriate halogen-containing quinazoline derivative (0.50 mmol): 2-(4-bromophenyl)4-(*N,N*-dimethylamino)quinazoline (**2a**), 2-(3-bromophenyl)-4-(*N,N*-dimethylamino)quinazoline (**2b**), 2-(2-bromophenyl)-4-(*N,N*-dimethylamino)quinazoline (**2c**), 6-bromo-4-(*N,N*-dimethylamino)-2-phenylquinazoline (**2f**), 7-bromo-4-(*N,N*-dimethylamino)-2-phenylquinazoline (**2g**), and bis[4-(tetramethyl-1,3,2-dioxaborolan-2-yl) phenyl] -1,3,4-oxadiazole (**7**) (125 mg; 0.28 mmol), Na_2_CO_3_ (224 mg; 2.11 mmol), Bu_4_NBr (14 mg; 0.042 mmol) were dissolved in toluene (6.6 mL) and H_2_O (3.4 mL) in glass tube reactor and the mixture was bubbled with Ar followed by addition of Pd(dppf)Cl_2_ (15 mg; 0.021 mmol). The glass tube reactor was sealed, and the reaction was stirred at 115 °C overnight. The solvent was evaporated, and the residue was portioned between DCM and H_2_O. The resulting mixture was extracted with DCM twice (2 × 10 mL). The final product was purified by column chromatography (silica gel, eluent: 0–10% EtOAc in DCM, 1–5% MeOH in DCM), followed by trituration with EtOAc to yield the appropriate symmetrical derivatives **9a-c** and **9e-f**.

*2,2′-(4′,4′′-(1,3,4-Oxadiazole-2,5-diyl)bis(biphenyl-4′,4-diyl))bis(N,N-dimethylquinazolin-4-amine)* (**9a**). The product was obtained as a pale yellow solid (106 mg, 70%); mp 271 °C (decomp.); R*_f_* = 0.47 (5% MeOH in DCM). IR (ATR): 2933, 1610, 1560, 1522, 1485, 1448, 1395, 1375, 1352, 1335, 1271, 1214, 1176, 1102, 1070, 1020, 1005, 954, 924, 866, 834, 798, 763, 739, 701, 680 cm^−1^. ^1^H-NMR (300 MHz, CDCl_3_) δ = 8.70 (d, *J* = 8.2 Hz, 4H), 8.27 (d, *J* = 8.4 Hz, 4H), 8.06 (d, *J* = 8.5 Hz, 2H), 7.98 (d, *J* = 8.2 Hz, 2H), 7.88 (d, *J* = 8.5 Hz, 4H), 7.80 (d, *J* = 8.4 Hz, 4H), 7.73 (t, *J* = 7.3 Hz, 2H), 7.40 (t, *J* = 7.7 Hz, 2H), 3.49 (s, 12H). ^13^C NMR (75 MHz, CDCl_3_) δ = 164.7, 164.0, 158.9, 153.1, 144.3, 141.2, 138.9, 132.2, 129.2, 128.8, 127.5, 127.1, 127.1, 125.7, 124.4, 122.9, 115.1, 42.0. HRMS (ESI/Q-TOF) *m/z*: [M + H]^+^ calcd for C_46_H_37_N_8_O 717.3090; found 717.3060. UV (MeOH): λ_max_ (ε) = 212.0 (12290), 269.0 (5150), 331.0 (12890).

*2,2′-(4′,4′′-(1,3,4-Oxadiazole-2,5-diyl)bis(biphenyl-4′,3-diyl))bis(N,N-dimethylquinazolin-4-amine)* (**9b**). The product was obtained as a cream solid (76 mg, 50%); mp 265–266 °C; R*_f_* = 0.48 (5% MeOH in DCM). IR (ATR): 3059, 2927, 1612, 1564, 1526, 1488, 1446, 1411, 1399, 1378, 13356, 1232, 1162, 1102, 1069, 1041, 1015, 961, 917, 888, 866, 845, 807, 791, 761, 752, 729, 709, 695, 680 cm^−1^. ^1^H-NMR (300 MHz, CDCl_3_) δ = 8.90 (t, *J* = 1.8 Hz, 2H), 8.62 (dt, *J* = 7.8, 1.2 Hz, 2H), 8.28 (d, *J* = 8.4 Hz, 4H), 8.06 (dd, *J* = 8.5, 1.3 Hz, 2H), 7.98 (dd, *J* = 8.5, 1.2 Hz, 2H), 7.92 (d, *J* = 8.4 Hz, 4H), 7.74 (tdd, *J* = 8.4, 6.6, 1.5 Hz, 4H), 7.61 (t, *J* = 7.7 Hz, 2H), 7.40 (ddd, *J* = 8.4, 6.6, 1.3 Hz, 2H), 3.48 (s, 12H). ^13^C-NMR (75 MHz, CDCl_3_) δ = 164.6, 164.0, 158.9, 153.0, 144.7, 139.8, 139.7, 132.1, 128.9, 128.8, 128.7, 128.3, 127.9, 127.4, 127.2, 125.6, 124.2, 122.7, 115.0, 41.9. HRMS (ESI/Q-TOF) *m/z*: [M + H]^+^ calcd for C_46_H_37_N_8_O 717.3090; found 717.3065. UV (MeOH): λ_max_ (ε) = 206.5 (10690), 210.5 (11190), 252.5 (9230), 267.5 (8940), 316.5 (11120).

*2,2′-(4′,4′′-(1,3,4-Oxadiazole-2,5-diyl)bis(biphenyl-4′,2-diyl))bis(N,N-dimethylquinazolin-4-amine)* (**9c**). The product was obtained as a colourless solid (91 mg, 60%); mp 165–166 °C; R*_f_* = 0.43 (5% MeOH in DCM). IR (ATR): 2927, 1732, 1611, 1562, 1522, 1499, 1444, 1398, 1375, 1351, 1240, 1219, 1142, 1095, 1071, 1047, 1021, 1005, 955, 879, 86, 807, 765, 739, 708, 638 cm^−1^. ^1^H-NMR (300 MHz, CDCl_3_) δ = 8.14–8.09 (m, 2H), 8.00–7.84 (m, 8H), 7.69 (ddd, *J* = 8.4, 6.9, 1.4 Hz, 2H), 7.57–7.44 (m, 6H), 7.42–7.33 (m, 6H), 2.94 (s, 12H). ^13^C-NMR (75 MHz, CDCl_3_) δ = 164.6, 162.8, 161.8, 152.7, 147.0, 140.4, 139.3, 132.1, 130.9, 130.5, 129.8, 129.1, 128.6, 128.2, 126.3, 125.5, 124.4, 121.5, 114.3, 41.4. HRMS (ESI/Q-TOF) *m/z*: [M + H]^+^ calcd for C_46_H_37_N_8_O 717.3090; found 717.3065. UV (MeOH): λ_max_ (ε) = 207.5 (10490), 249.0 (54350), 314.5 (61870).

*6,6′-(4,4′-(1,3,4-Oxadiazole-2,5-diyl)bis(4,1-phenylene))bis(N,N-dimethylquinazolin-4-amine)* (**9f**). The product was obtained as a yellow solid(79 mg, 52%); mp 378–330 °C; R*_f_* = 0.70 (5% MeOH in DCM). IR (ATR): 3058, 2881, 2170, 1608, 1584, 1553, 1520, 1485, 1449, 1413, 1390, 1348, 1324, 1240, 1191, 1168, 1105, 1066, 1026, 1013, 958, 891, 829, 803, 779, 749, 708, 672 cm^−1^. ^1^H-NMR (300 MHz, CDCl_3_) δ = 8.66–8.57 (m, 4H), 8.37–8.27 (m, 6H), 8.16–8.00 (m, 4H), 7.88 (d, *J* = 8.2 Hz, 4H), 7.52 (d, *J* = 7.0 Hz, 6H), 3.55 (s, 12H). ^13^C-NMR (75 MHz, CDCl_3_) δ = 164.8, 160.3, 159.8, 153.0, 143.9, 138.8, 135.5, 131.2, 130.3, 129.6, 129.3, 128.6, 128.4, 127.1, 124.1, 123.0, 115.3, 42.08. HRMS (ESI/Q-TOF) *m/z*: [M + H]^+^ calcd for C_46_H_37_N_8_O 717.3090; found 717.3088. UV (DCM): λ_max_ (ε) = 228.5 (55720), 292.0 (50750), 361.0 (80530).

*7,7′-(4,4′-(1,3,4-Oxadiazole-2,5-diyl)bis(4,1-phenylene))bis(N,N-dimethylquinazolin-4-amine)* (**9g**). The product was obtained as a beige solid(98 mg, 65%); mp 257–258 °C (decomp.); R*_f_* = 0.45 (5% MeOH in DCM). IR (ATR): 2881, 1614, 1559, 1533, 1474, 1453, 1429, 1382, 1345, 1201, 1167, 1103, 1068, 1026, 1014, 955, 911, 890, 847, 823, 798, 777, 746, 704 cm^−1^. ^1^H-NMR (300 MHz, CDCl_3_) δ = 8.67–8.57 (m, 4H), 8.36–8.23 (m, 6H), 8.16 (d, *J* = 8.7 Hz, 2H), 7.96 (d, *J* = 8.1 Hz, 4H), 7.70 (dd, *J* = 8.7, 1.9 Hz, 2H), 7.59–7.46 (m, 6H), 3.52 (s, 12H). ^13^C-NMR (75 MHz, CDCl_3_) δ = 164.5, 163.6, 159.9, 153.5, 143.1, 143.0, 138.7, 130.2, 128.4, 128.3, 128.0, 127.6, 126.7, 126.4, 123.5, 122.9, 114.4, 41.8. HRMS (ESI/Q-TOF) *m/z*: [M + H]^+^ calcd for C_46_H_37_N_8_O 717.3090; found 717.3096. UV (DCM): λ_max_ (ε) = 222.0 (37810), 276.0 (50200), 329.0 (110400).

## 4. Conclusions

In conclusion, we have synthesized two series of novel, highly-conjugated, unsymmetrical and symmetrical quinazolinylphenyl-1,3,4-oxadiazole compounds. We demonstrated a simple and efficient synthetic methodology that involves Suzuki cross-coupling reactions between bromine-containing 4-(N,N-dimethylamine)-2-phenylquinazoline derivatives and 2,5-diphenyl-1,3,4-oxadiazole-bearing boronic esters in the presence of a palladium catalyst under phase transfer catalytic conditions. The prepared boronic acid pinacol ester and diboronic acid bis(pinacol) ester of 2,5-diphenyl-1,3,4-oxadiazole were effective precursors for the described Suzuki cross-coupling reactions. Both the boronic esters **6**, **7** and final products **8f**, **9f**, wherein the 1,3,4-oxadiazole moiety is connected to quinazoline ring by a 1,4-phenylene linker at the 6 position, displayed strong fluorescence emission and high quantum yields, making these compounds potentially useful building blocks for optoelectronic applications.

## Figures and Tables

**Table 1:** Initial optimization for the cross-coupling reaction to obtain **8a**.

Entry	Catalyst	Base	Solvent	PTC Catalyst	Yield [%] ^a^
1	Pd(PPh_3_)_4_	Na_2_CO_3_	Toluene/water	Aliquat 336	63
2	Pd(PPh_3_)_4_	Na_2_CO_3_	Toluene/water	BnEt_3_NCl	61
3	Pd(PPh_3_)_4_	Na_2_CO_3_	Toluene/water	Bu_4_NCl	65
4	Pd(PPh_3_)_4_	Na_2_CO_3_	Toluene/water	Bu_4_NBr	68
5	Pd(t-Bu_3_P)_2_	Na_2_CO_3_	Toluene/water	Bu_4_NBr	68
6	Pd(dppf)Cl_2_	Na_2_CO_3_	Toluene/water	Bu_4_NBr	85
7	Pd(PPh_3_)_2_Cl_2_	Na_2_CO_3_	Toluene/water	Bu_4_NBr	77
8	Pd(dppf)Cl_2_	K_2_CO_3_	Toluene/water	Bu_4_NBr	60
9	Pd(dppf)Cl_2_	t-BuOK	Toluene/water	Bu_4_NBr	70
10	Pd(dppf)Cl_2_	AcONa	Toluene/water	Bu_4_NBr	66
11	Pd(dppf)Cl_2_	Na_2_CO_3_	Dioxane	-	74
12	Pd(dppf)Cl_2_	Na_2_CO_3_	EtOH	-	77
13	Pd(dppf)Cl_2_	Na_2_CO_3_	DME	-	61
14	Pd(dppf)Cl_2_	Na_2_CO_3_	DMF	-	64

^a^ Yield with respect to starting quinazoline **2a**. Conditions: sealed tube, oil bath 115 °C, overnight.

**Table 2:** Synthesis of unsymmetrical quinazolinylphenyl-1,3,4-oxadiazole derivatives (**8a-c, 8f‑g**) using Suzuki cross-coupling reactions.

Entry	Substrate		Product		Yield [%] ^a^
1.	**2a**	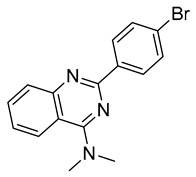	**8a**	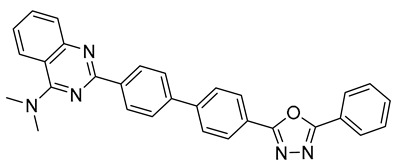	82 ^a^
2.	**2b**	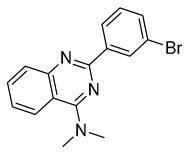	**8b**	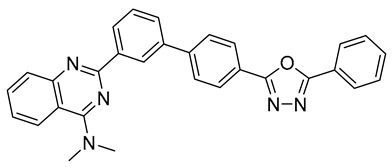	74 ^a^
3.	**2c**	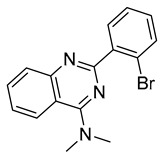	**8c**	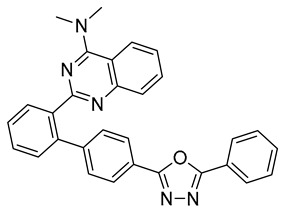	86 ^a^
4.	**2d**	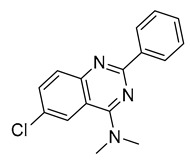	No reaction ^a,b^		-
5.	**2e**	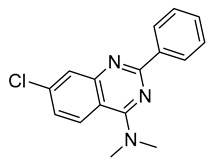	No reaction ^a,b^		-
6.	**2f**	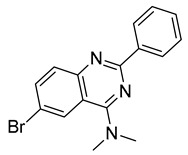	**8f**	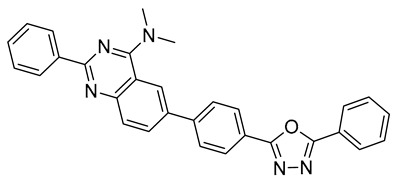	75 ^a^
7.	**2g**	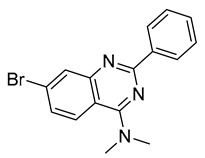	**8g**	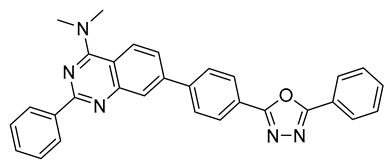	72 ^a^

^a^ Yield with respect to starting quinazoline derivative **2a-c**, **2f-g**. ^b^ For **2d** and **2f** additional experiments in MW, 120 °C, 60 min.

**Table 3:** Synthesis of symmetrical 2,5-bis(quinazolinylphenyl)-1,3,4-oxadiazole derivatives (**9a-c**, **9f-g**) using Suzuki cross-coupling reactions.

Entry	Substrate	Product		Yield [%] ^a^
1.	**2a**	**9a**	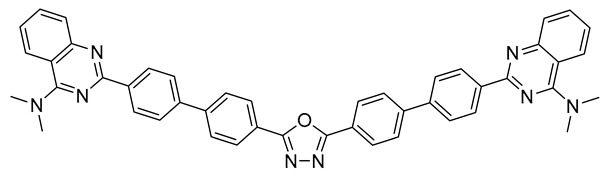	70
2.	**2b**	**9b**	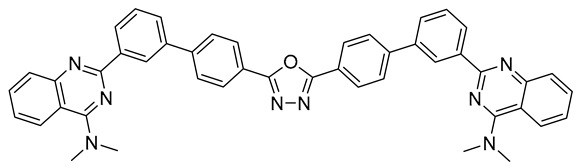	50
3.	**2c**	**9c**	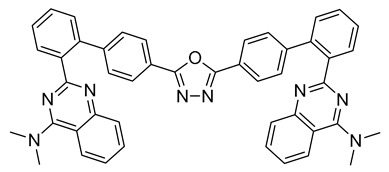	60
4.	**2d**	No reaction		-
5.	**2e**	No reaction		-
6.	**2f**	**9f**	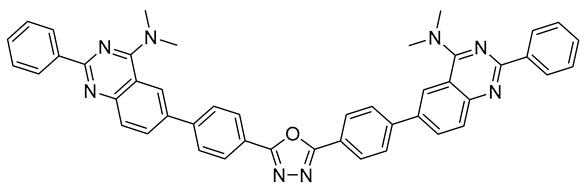	52
7.	**2g**	**9g**	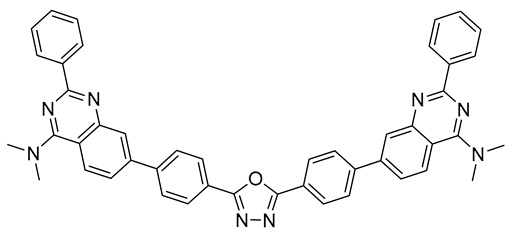	65

^a^ Yield with respect to starting quinazoline derivative **2a-c**, **2f-g**.

**Table 4:** Absorption and fluorescence spectral parameters of the investigated compounds, **6**, **7**, **8a-c**, **8f-g**, **9a-c**, **9f-g**.

Entry	Compound	Absorption Maximum λ_max_ [nm] (ε.10^−4^ m^3^/(mol.cm)])	Excitation Wavelength λ_ex_ [nm]	Emission Wavelength λ_em_ [nm]	Stokes Shift ^a^ ∆ [nm]	Quantum Yield ^b^ Φ_f_
1.	**6**	291 (3.02)	290	343 (max), 355	52	0.68
2.	**7**	298 (2.97)	300	333, 349 (max), 364	51	0.91
3.	**8a**	329 (4.98)	330	414	85	0.01
4.	**8b**	305 (4.37)	310	365 (max), 385	60	0.02
5.	**8c**	302 (2.94)	305	366 (max), 384	64	0.13
6.	**8f**	297 (5.63)	300	429	131	0.61
7.	**8g**	314 (7.44)	315	419	105	0.08
8.	**9a**	342 (6.91)	340	424	82	0.05
9.	**9b**	320 (8.46)	320	378 (max), 397	58	0.01
10.	**9c**	319 (4.78)	320	427	108	0.01
11.	**9f**	292 (5.07)	295	424	70	0.98
12.	**9g**	329 (11.04)	330	421	92	0.06

^a^ Stokes shift were calculated from the equation Δ = λ_em_ − λ_max_; ^b^ the quantum yields Φ_f_ were calculated at λ_ex_ = 355 nm in cyclohexane in comparison to DPA (Φ_f_ = 0.97) and at λ_ex_ = 320 nm in *n*-hexane in comparison to DPB (Φ_f_ = 0.35)—the mean value is presented.

## Supplementary Materials

Copies of the ^1^H-NMR and ^13^C-NMR spectra of the compounds are available in the online Supplementary Materials.
