# Supplementary material for: Efficient Synthesis of Novel 1,3,4-Oxadiazoles Bearing a 4-N,N-Dimethylaminoquinazoline Scaffold via Palladium-Catalyzed Suzuki Cross-Coupling Reactions
Source: Molecules. 2020 Nov 5;25(21):5150. doi: 10.3390/molecules25215150 (PMC7663961; doi:10.3390/molecules25215150)

Efficient Synthesis of Novel 1,3,4-Oxadiazoles Bearing a 4-*N*,*N*-Dimethylaminoquinazoline Scaffold via Palladium-Catalyzed Suzuki Cross-Coupling Reactions

Barbara Wołek, Mateusz Werłos, Magdalena Komander, Agnieszka Kudelko

Supplementary Materials

Table of Contents

^{1}H NMR spectrum of 2-(3-Bromophenyl)-4-(*N,N*-dimethylamino)quinazoline (**2b**): 3

^{13}C NMR spectrum of 2-(3-Bromophenyl)-4-(*N,N*-dimethylamino)quinazoline (**2b**): 4

^{1}H NMR spectrum of 2-(2-Bromophenyl)-4-(*N,N*-dimethylamino)quinazoline (**2c**): 5

^{13}C NMR spectrum of 2-(2-Bromophenyl)-4-(*N,N*-dimethylamino)quinazoline (**2c**): 6

^{1}H NMR spectrum of 7-Chloro-4-(*N,N*-dimethylamino)-2-phenylquinazoline (**2e**): 7

^{13}C NMR spectrum of 7-Chloro-4-(*N,N*-dimethylamino)-2-phenylquinazoline (**2e**): 8

^{1}H NMR spectrum of 7-Bromo-4-(*N,N*-dimethylamino)-2-phenylquinazoline (**2g**): 9

^{13}C NMR spectrum of 7-Bromo-4-(*N,N*-dimethylamino)-2-phenylquinazoline (**2g**): 10

^{1}H NMR spectrum of 2-Phenyl-5-[4-(tetramethyl-1,3,2-dioxaborolan-2-yl)phenyl]-1,3,4-oxadiazole (**6**): 11

^{13}C NMR spectrum of 2-Phenyl-5-[4-(tetramethyl-1,3,2-dioxaborolan-2-yl)phenyl]-1,3,4-oxadiazole (**6**): 12

^{1}H NMR spectrum of bis[4-(Tetramethyl-1,3,2-dioxaborolan-2-yl)phenyl]-1,3,4-oxadiazole (**7**): 13

^{13}C NMR spectrum of bis[4-(Tetramethyl-1,3,2-dioxaborolan-2-yl)phenyl]-1,3,4-oxadiazole (**7**): 14

^{1}H NMR spectrum of *N,N*-Dimethyl-2-(4'-(5-phenyl-1,3,4-oxadiazol-2-yl)biphenyl-4-yl)quinazolin-4-amine (**8a**): 15

^{13}C NMR spectrum of *N,N*-Dimethyl-2-(4'-(5-phenyl-1,3,4-oxadiazol-2-yl)biphenyl-4-yl)quinazolin-4-amine (**8a**): 16

^{1}H NMR spectrum of *N,N*-Dimethyl-2-(4'-(5-phenyl-1,3,4-oxadiazol-2-yl)biphenyl-3-yl)quinazolin-4-amine (**8b**): 17

^{13}C NMR spectrum of *N,N*-Dimethyl-2-(4'-(5-phenyl-1,3,4-oxadiazol-2-yl)biphenyl-3-yl)quinazolin-4-amine (**8b**): 18

^{1}H NMR spectrum of *N,N*-Dimethyl-2-(4'-(5-phenyl-1,3,4-oxadiazol-2-yl)biphenyl-2-yl)quinazolin-4-amine (**8c**): 19

^{13}C NMR spectrum of *N,N*-Dimethyl-2-(4'-(5-phenyl-1,3,4-oxadiazol-2-yl)biphenyl-2-yl)quinazolin-4-amine (**8c**): 20

^{1}H NMR spectrum of *N,N*-Dimethyl-2-phenyl-6-(4-(5-phenyl-1,3,4-oxadiazol-2-yl)phenyl)quinazolin-4-amine (8f): 21

^{13}C NMR spectrum of *N,N*-Dimethyl-2-phenyl-6-(4-(5-phenyl-1,3,4-oxadiazol-2-yl)phenyl)quinazolin-4-amine (**8f**): 22

^{1}H NMR spectrum of *N,N*-Dimethyl-2-phenyl-7-(4-(5-phenyl-1,3,4-oxadiazol-2-yl)phenyl)quinazolin-4-amine (**8g**): 23

^{13}C NMR spectrum of *N*,*N*-Dimethyl-2-phenyl-7-(4-(5-phenyl-1,3,4-oxadiazol-2-yl)phenyl)quinazolin-4-amine (**8g**): 24

^{1}H NMR spectrum of 2,2'-(4',4''-(1,3,4-Oxadiazole-2,5-diyl)bis(biphenyl-4',4-diyl))bis(*N*,*N*-dimethylquinazolin-4-amine) (**9a**): 25

^{13}C NMR spectrum of 2,2'-(4',4''-(1,3,4-Oxadiazole-2,5-diyl)bis(biphenyl-4',4-diyl))bis(*N*,*N*-dimethylquinazolin-4-amine) (**9a**): 26

^{1}H NMR spectrum of 2,2'-(4',4''-(1,3,4-Oxadiazole-2,5-diyl)bis(biphenyl-4',3-diyl))bis(*N*,*N*-dimethylquinazolin-4-amine) (**9b**): 27

^{13}C NMR spectrum of 2,2'-(4',4''-(1,3,4-Oxadiazole-2,5-diyl)bis(biphenyl-4',3-diyl))bis(*N*,*N*-dimethylquinazolin-4-amine) (**9b**): 28

^{1}H NMR spectrum of 2,2'-(4',4''-(1,3,4-Oxadiazole-2,5-diyl)bis(biphenyl-4',2-diyl))bis(*N*,*N*-dimethylquinazolin-4-amine) (**9c**): 29

^{13}C NMR spectrum of 2,2'-(4',4''-(1,3,4-Oxadiazole-2,5-diyl)bis(biphenyl-4',2-diyl))bis(*N*,*N*-dimethylquinazolin-4-amine) (**9c**): 30

^{1}H NMR spectrum of 6,6'-(4,4'-(1,3,4-Oxadiazole-2,5-diyl)bis(4,1-phenylene))bis(*N*,*N*-dimethylquinazolin-4-amine) (**9f**): 31

^{13}C NMR spectrum of 6,6'-(4,4'-(1,3,4-Oxadiazole-2,5-diyl)bis(4,1-phenylene))bis(*N*,*N*-dimethylquinazolin-4-amine) (**9f**): 32

^{1}H NMR spectrum of 7,7'-(4,4'-(1,3,4-Oxadiazole-2,5-diyl)bis(4,1-phenylene))bis(*N*,*N*-dimethylquinazolin-4-amine) (**9g**): 33

^{13}C NMR spectrum of 7,7'-(4,4'-(1,3,4-Oxadiazole-2,5-diyl)bis(4,1-phenylene))bis(*N*,*N*-dimethylquinazolin-4-amine) (**9g**): 34

^{1}H NMR spectrum of 2-(3-Bromophenyl)-4-(*N*,*N*-dimethylamino)quinazoline (2b):

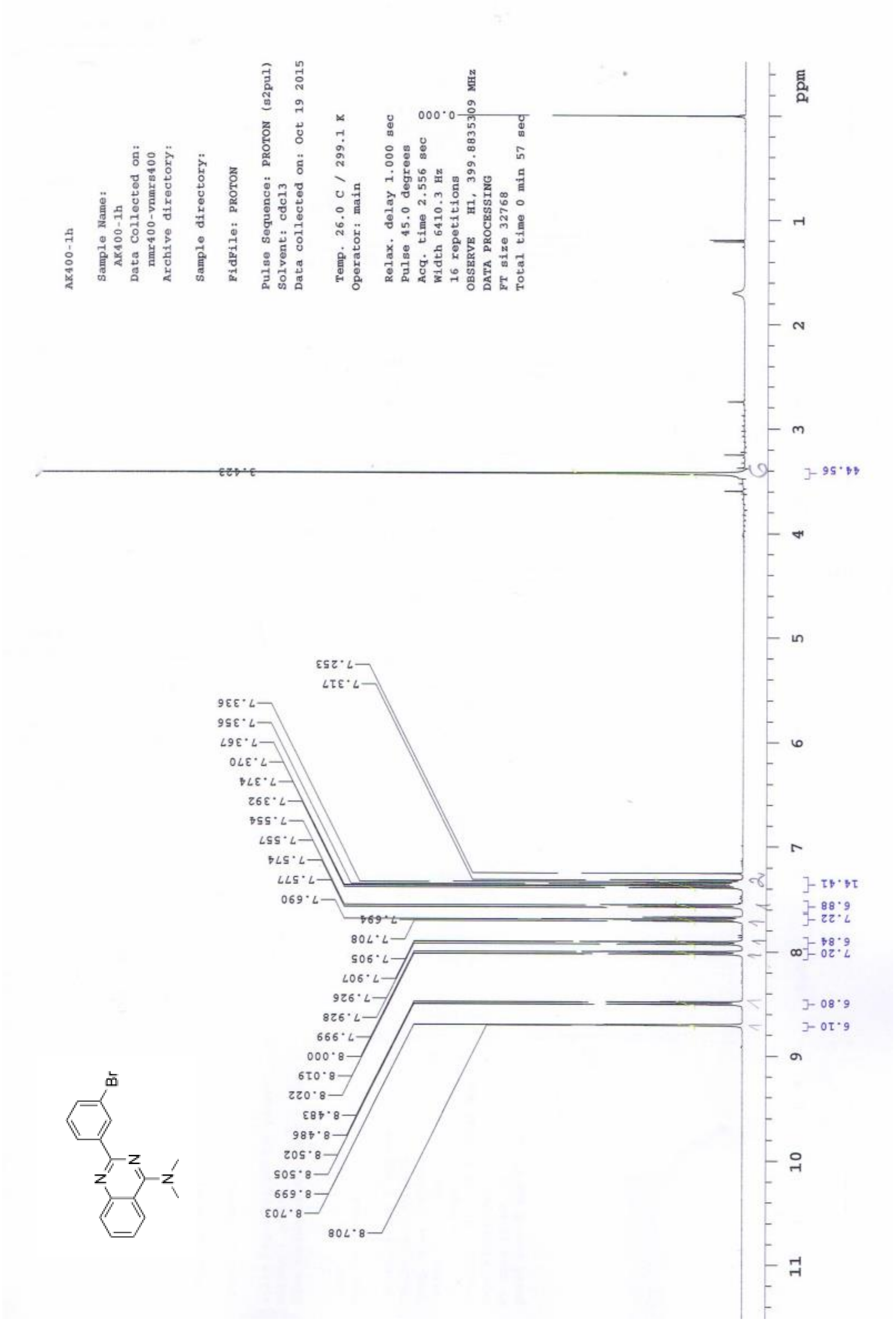

^{13}C NMR spectrum of 2-(3-Bromophenyl)-4-(*N*,*N*-dimethylamino)quinazoline (2b):

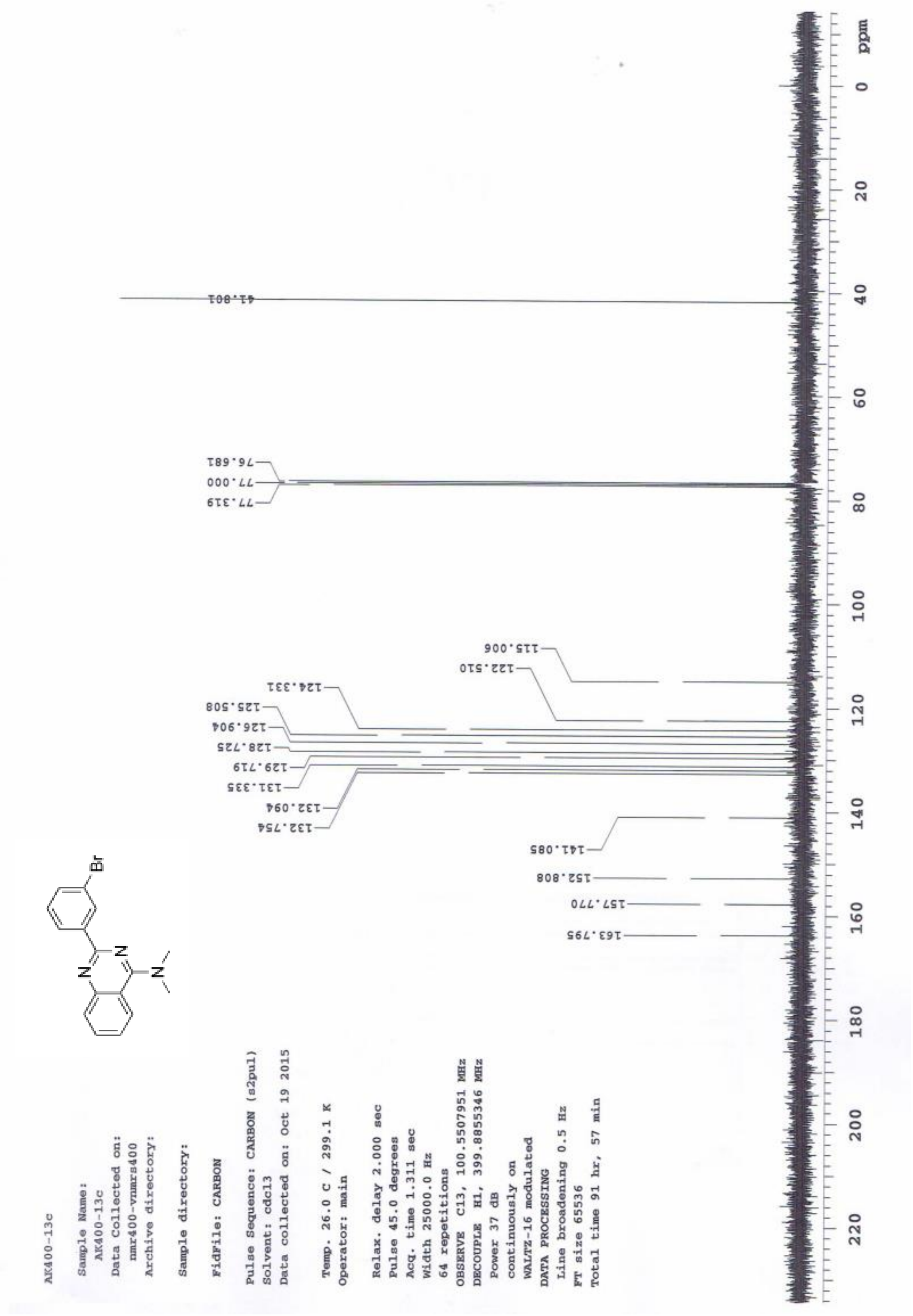

^{1}H NMR spectrum of 2-(2-Bromophenyl)-4-(*N*,*N*-dimethylamino)quinazoline (2c):

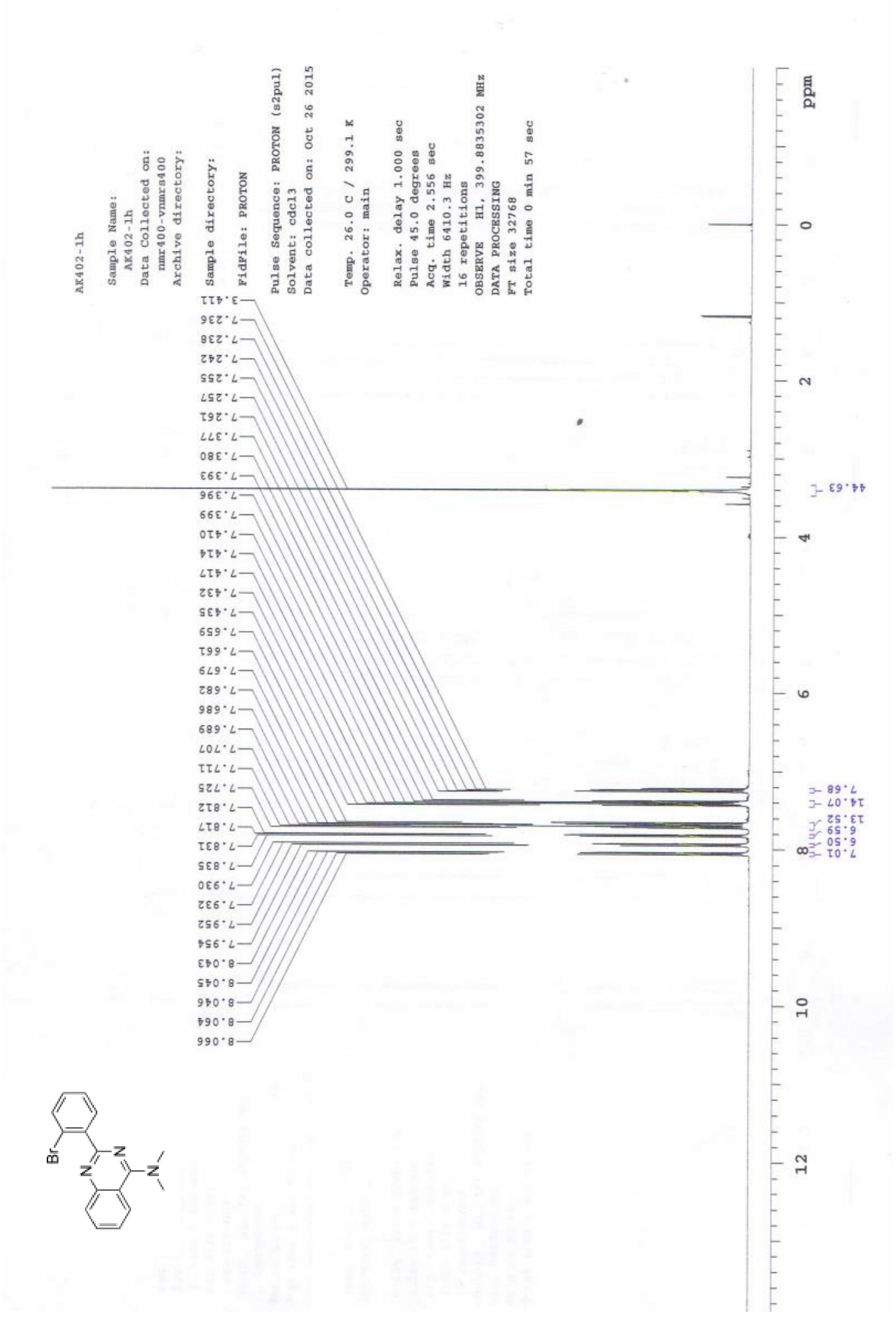

^{13}C NMR spectrum of 2-(2-Bromophenyl)-4-(*N*,*N*-dimethylamino)quinazoline (2c):

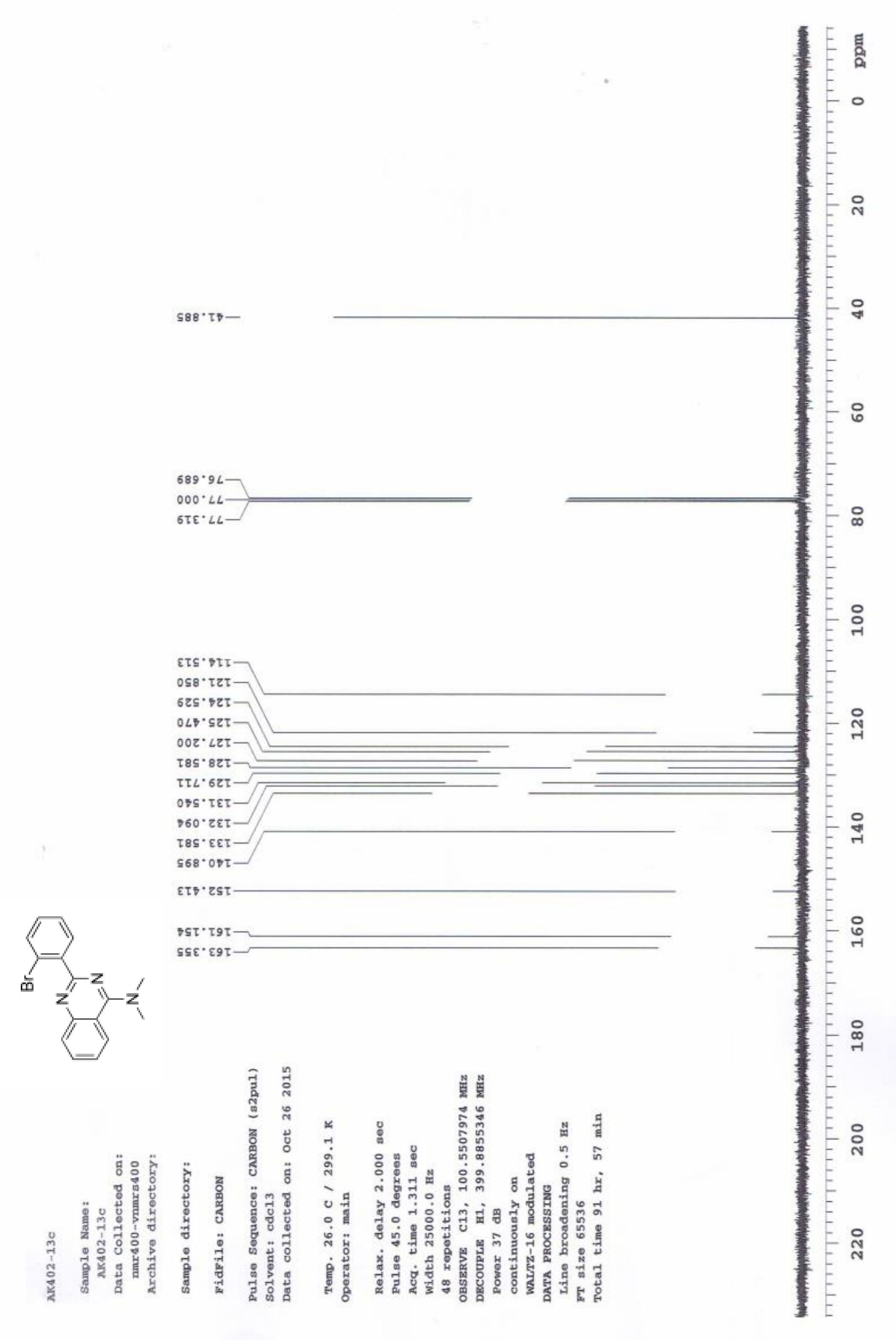

^{1}H NMR spectrum of 7-Chloro-4-(*N*,*N*-dimethylamino)-2-phenylquinazoline (2e):

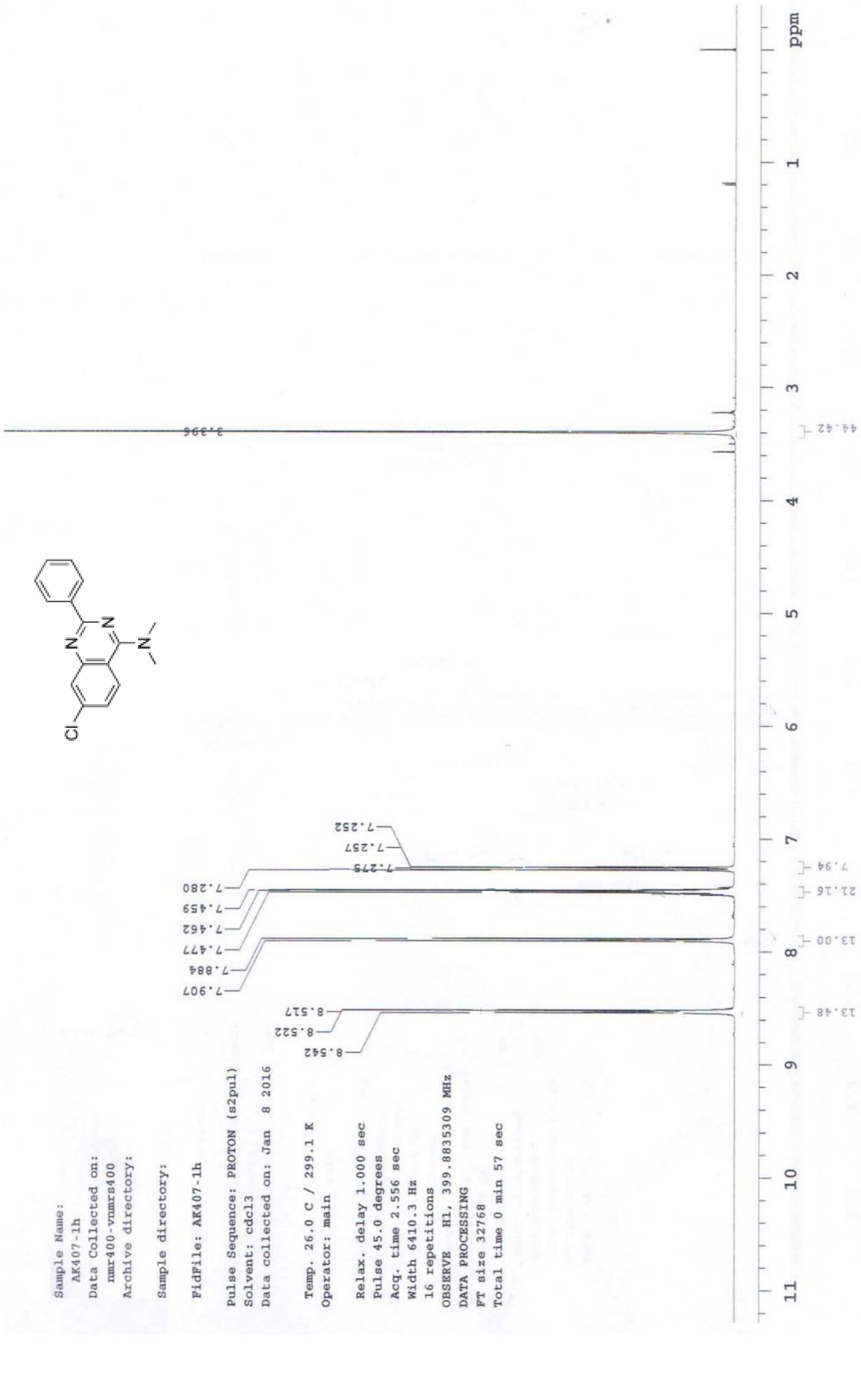

^{13}C NMR spectrum of 7-Chloro-4-(*N*,*N*-dimethylamino)-2-phenylquinazoline (2e):

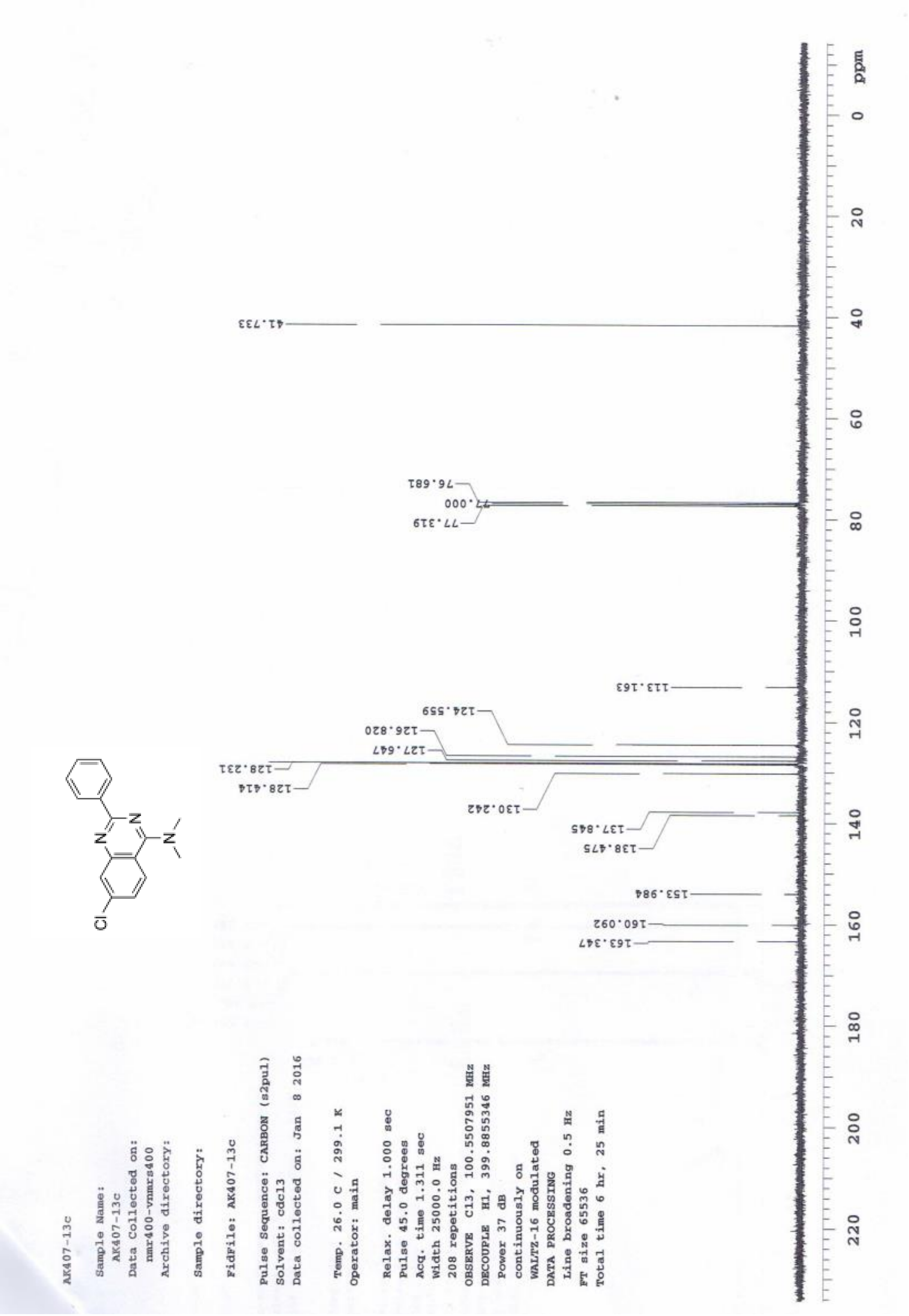

^{1}H NMR spectrum of 7-Bromo-4-(*N*,*N*-dimethylamino)-2-phenylquinazoline (2g):

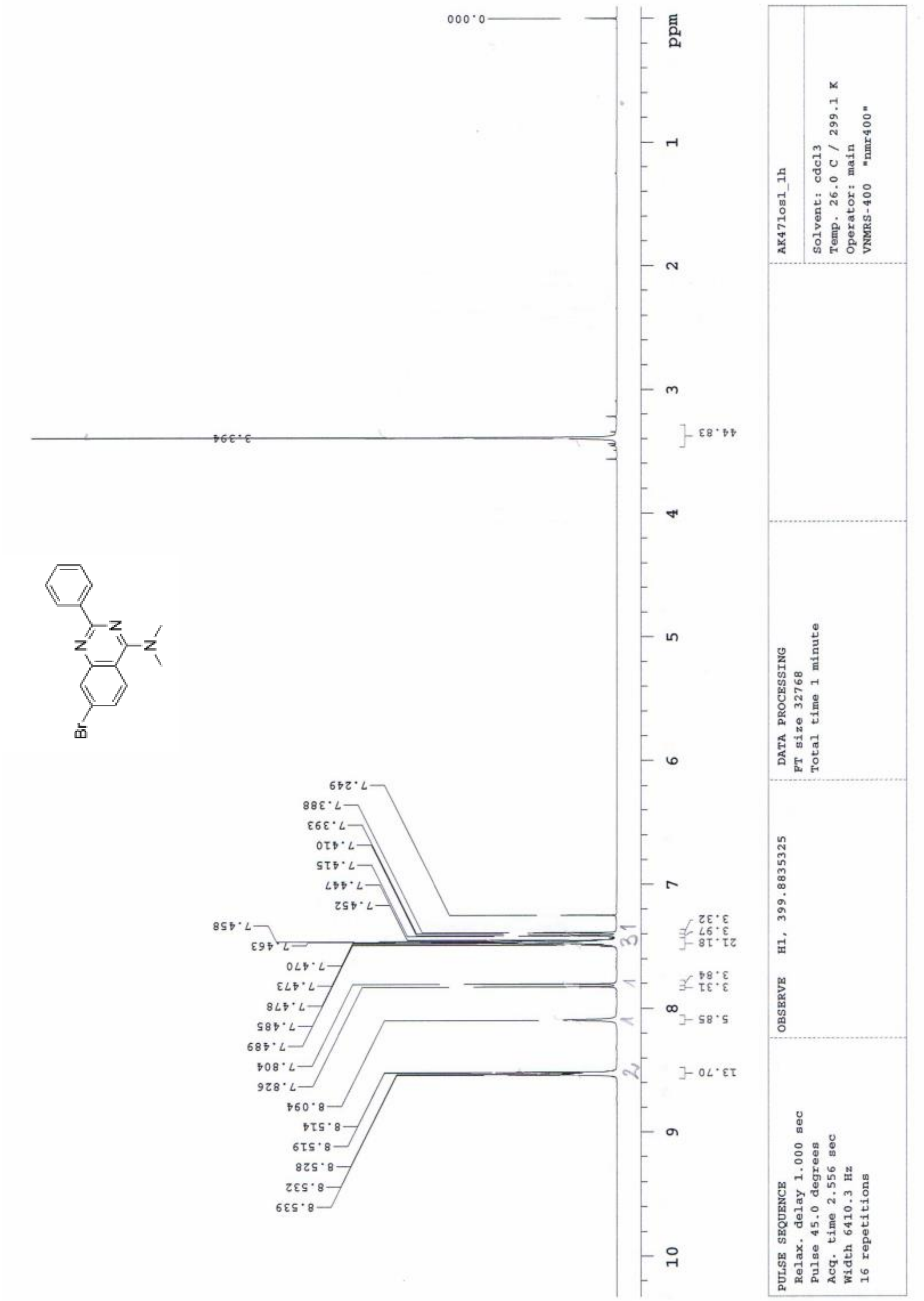

^{13}C NMR spectrum of 7-Bromo-4-(*N*,*N*-dimethylamino)-2-phenylquinazoline (2g):

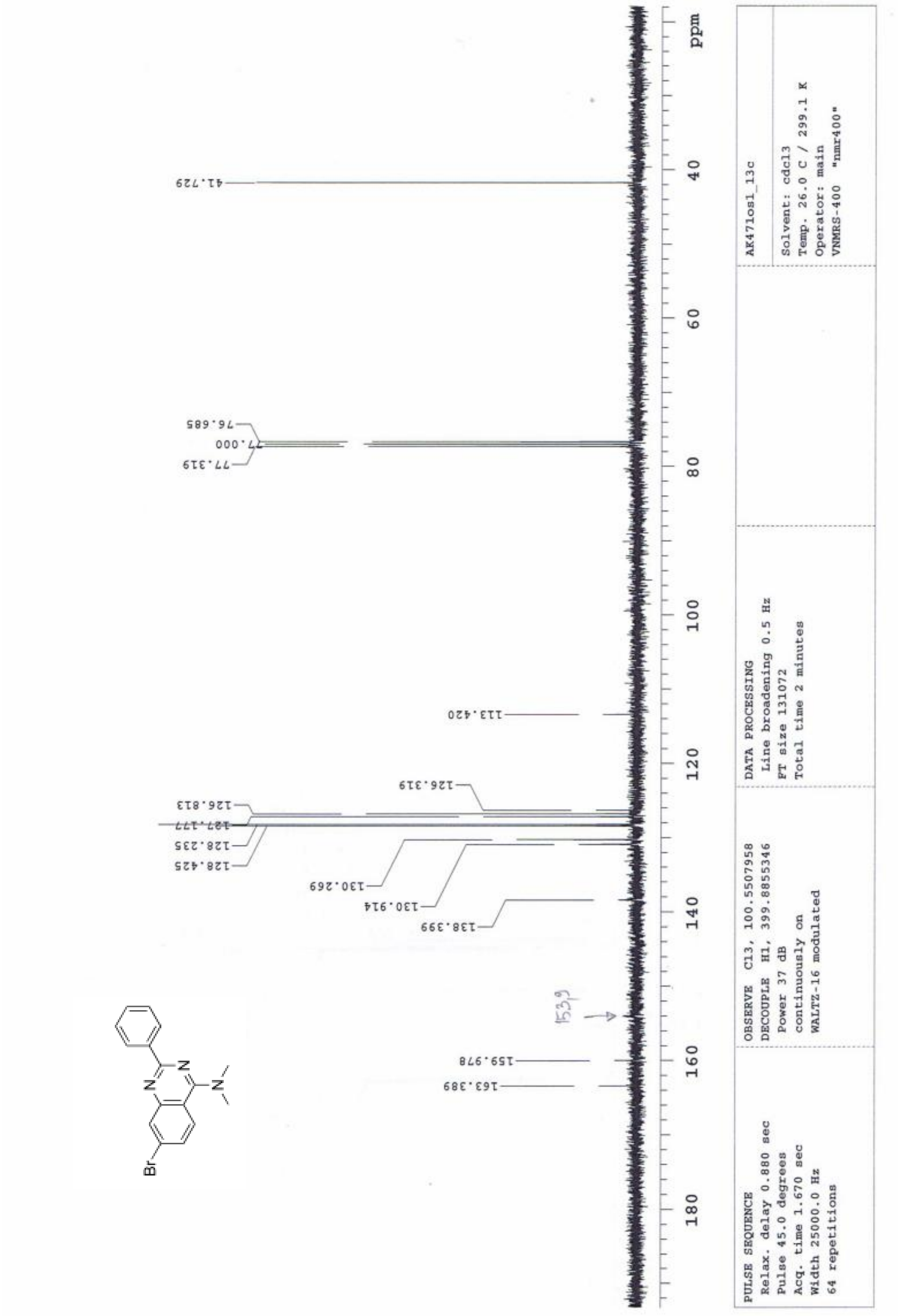

^{1}H NMR spectrum of 2-Phenyl-5-[4-(tetramethyl-1,3,2-dioxaborolan-2-yl)phenyl]-1,3,4-oxadiazole (6):

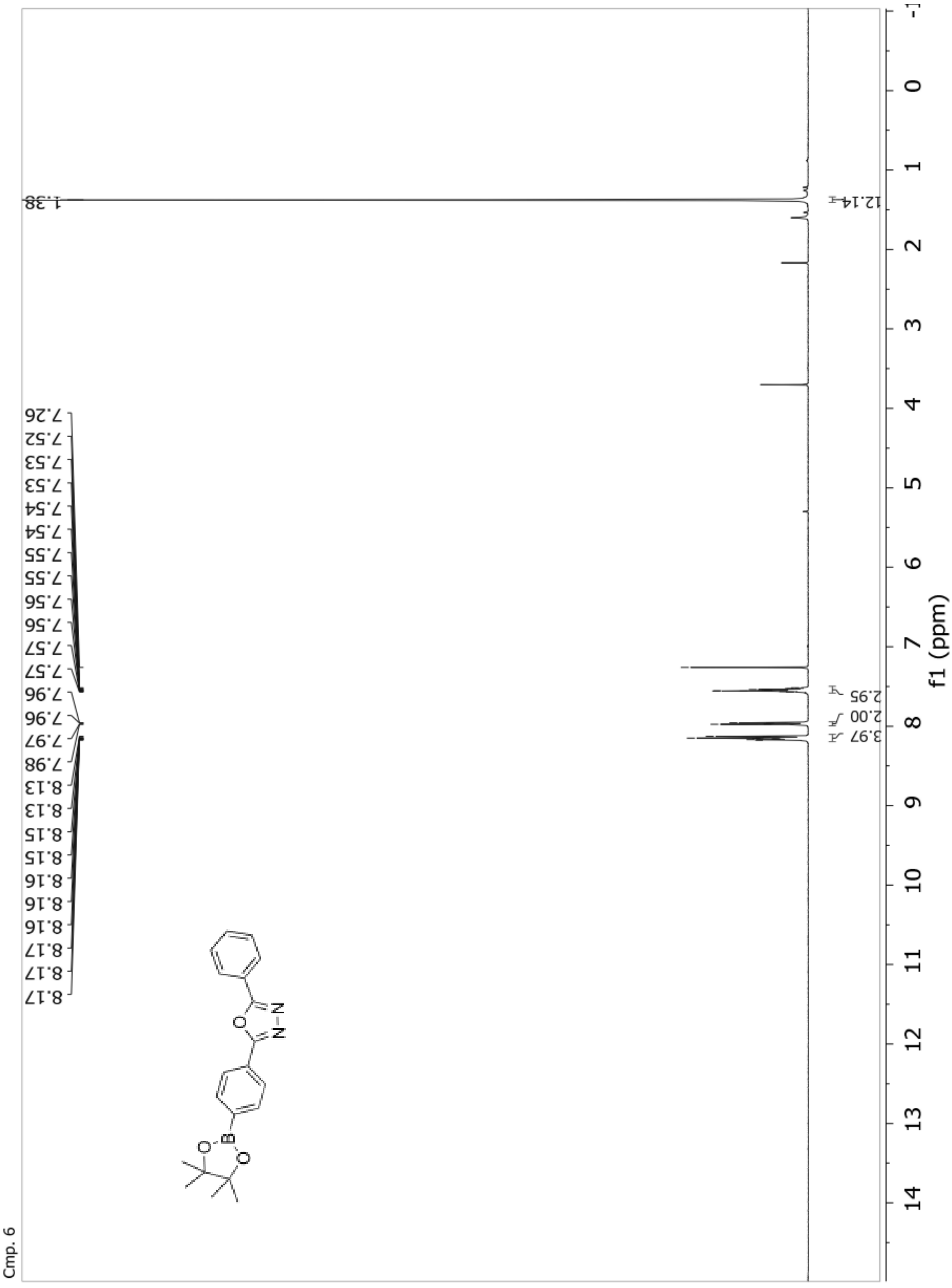

^{13}C NMR spectrum of 2-Phenyl-5-[4-(tetramethyl-1,3,2-dioxaborolan-2-yl)phenyl]-1,3,4-oxadiazole (6):

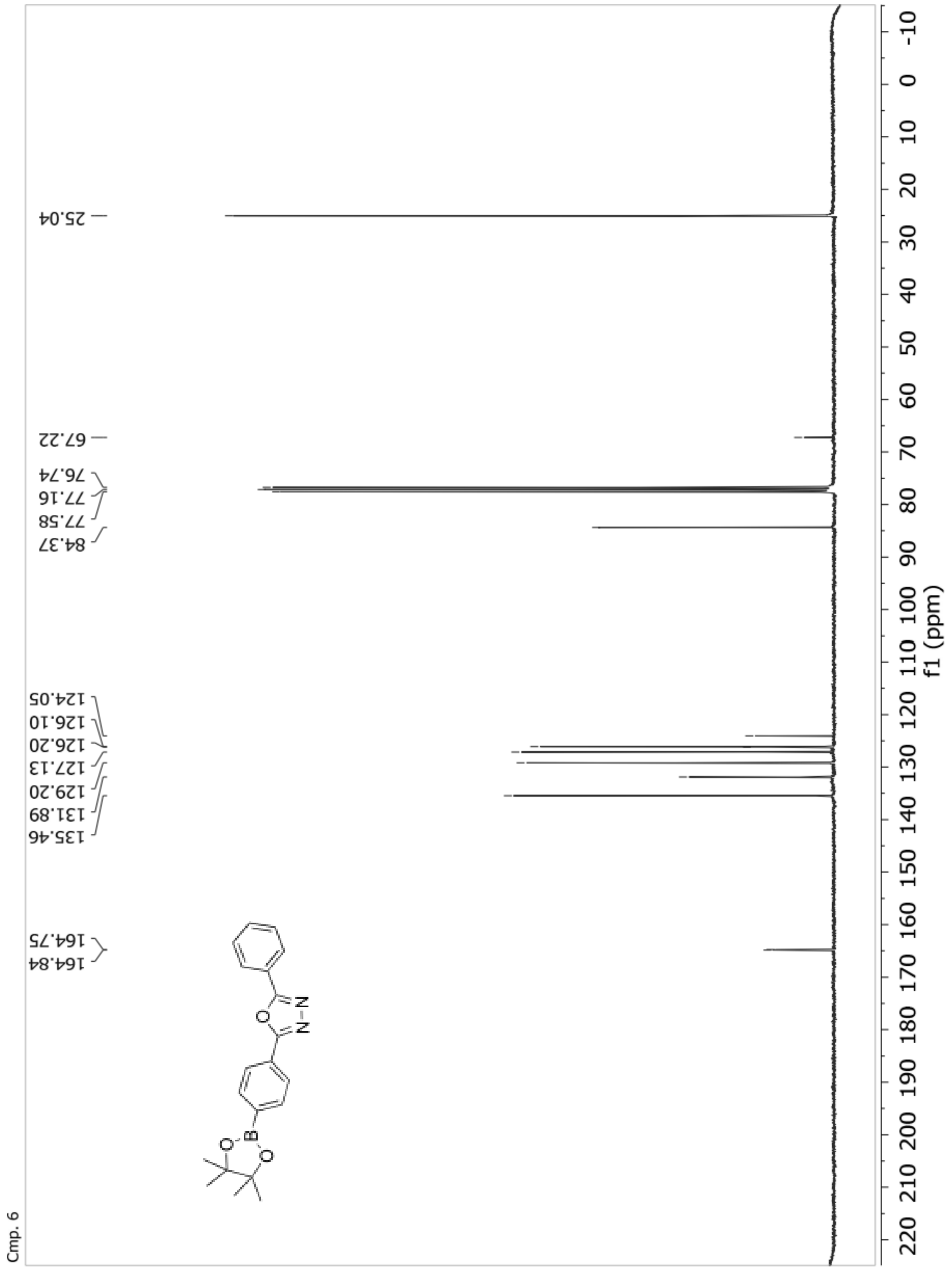

^{1}H NMR spectrum of bis[4-(Tetramethyl-1,3,2-dioxaborolan-2-yl)phenyl]-1,3,4-oxadiazole (7):

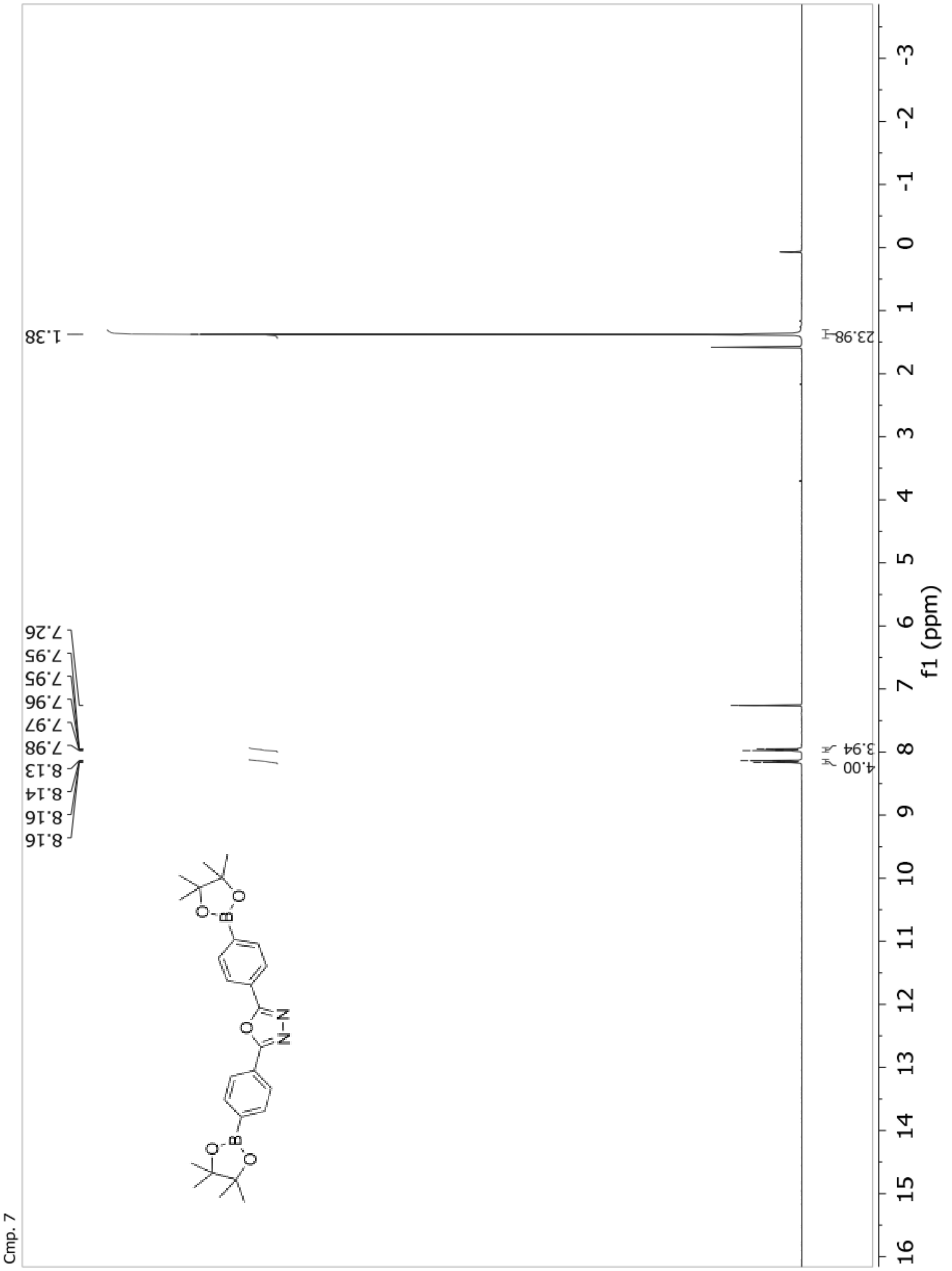

^{13}C NMR spectrum of bis[4-(Tetramethyl-1,3,2-dioxaborolan-2-yl)phenyl]-1,3,4-oxadiazole (7):

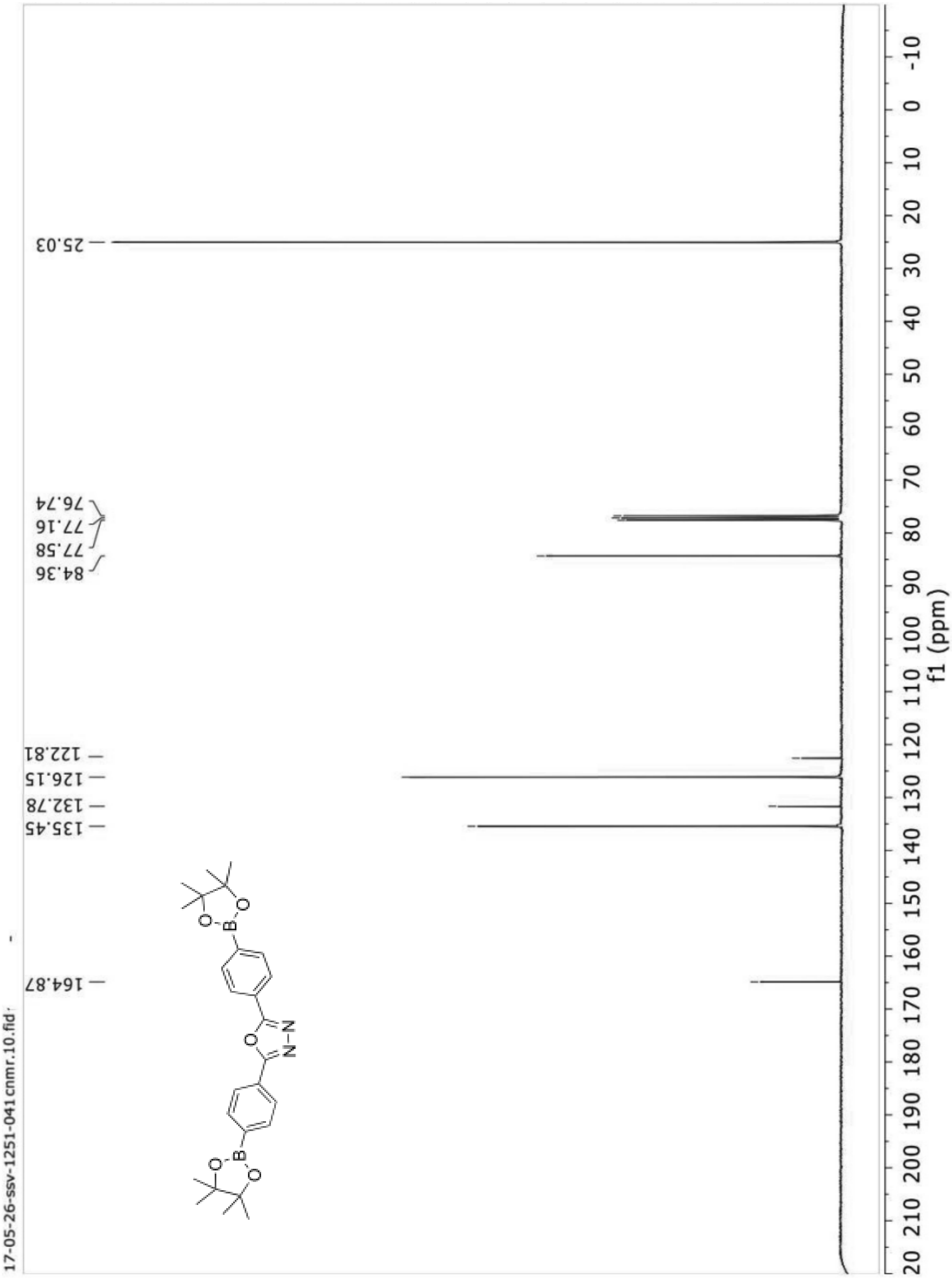

^{1}H NMR spectrum of *N*,*N*-Dimethyl-2-(4'-(5-phenyl-1,3,4-oxadiazol-2-yl)biphenyl-4-yl)quinazolin-4-amine (8a):

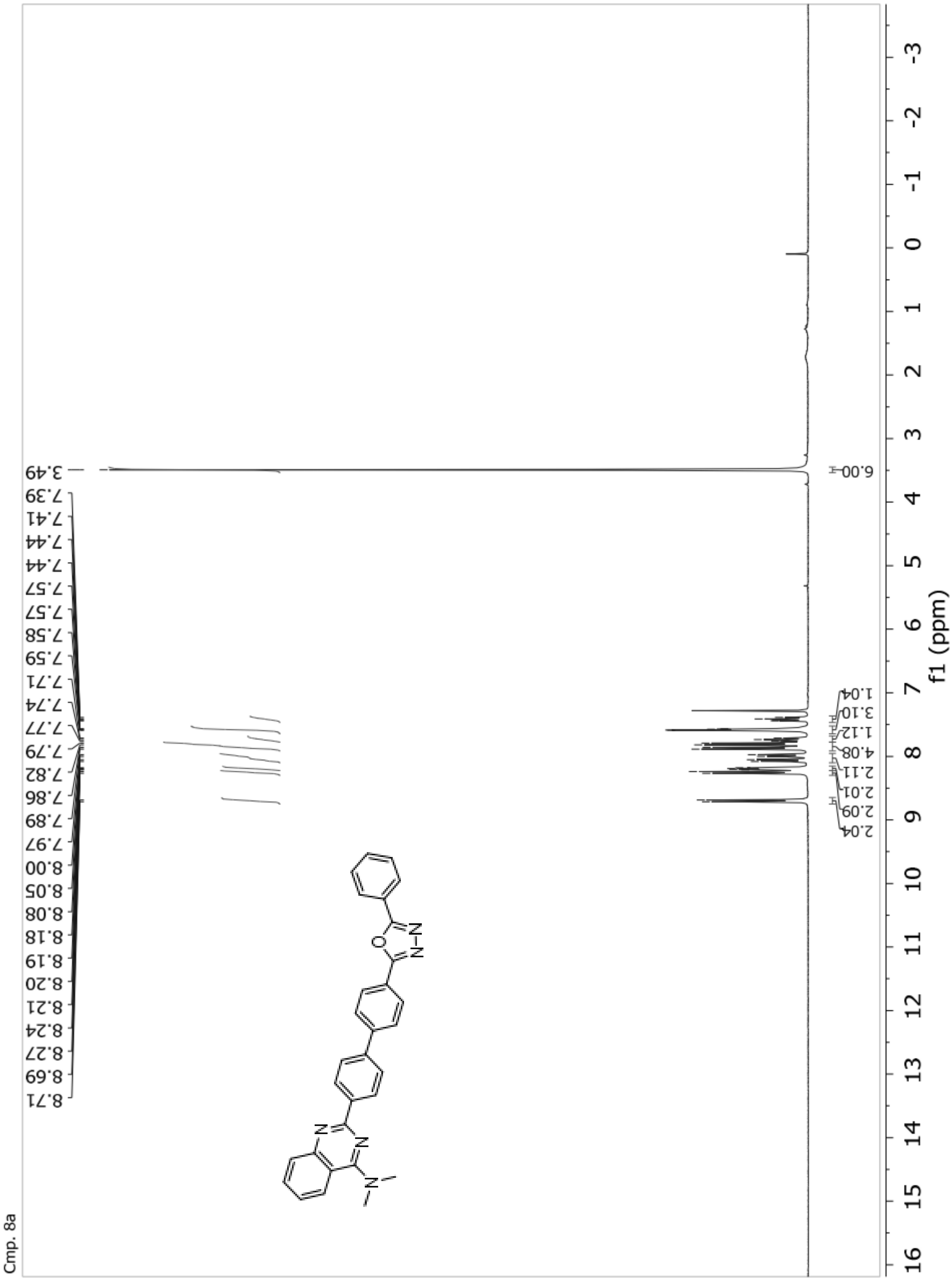

^{13}C NMR spectrum of *N*,*N*-Dimethyl-2-(4'-(5-phenyl-1,3,4-oxadiazol-2-yl)biphenyl-4-yl)quinazolin-4-amine (8a):

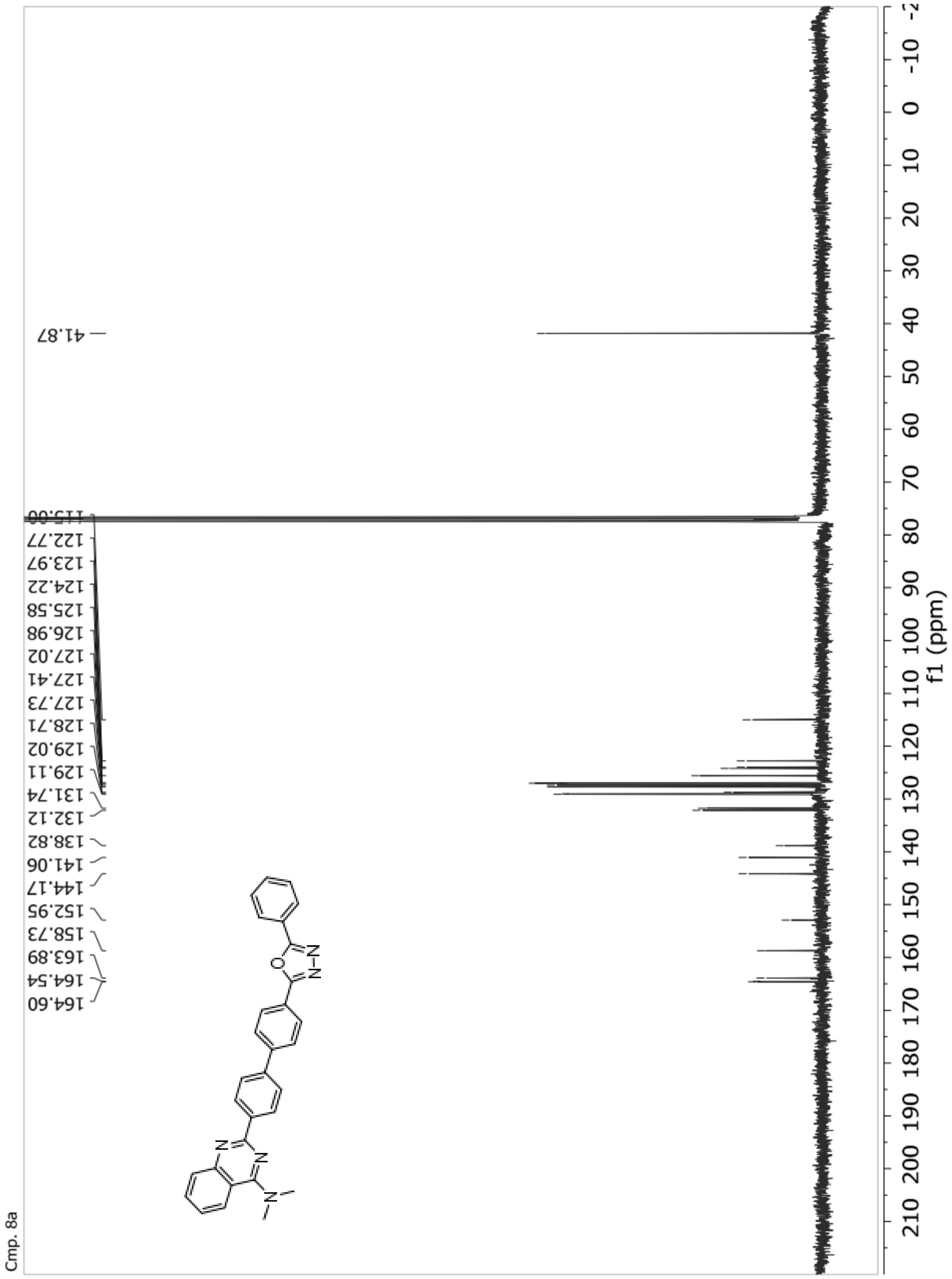

^{1}H NMR spectrum of *N*,*N*-Dimethyl-2-(4'-(5-phenyl-1,3,4-oxadiazol-2-yl)biphenyl-3-yl)quinazolin-4-amine (8b):

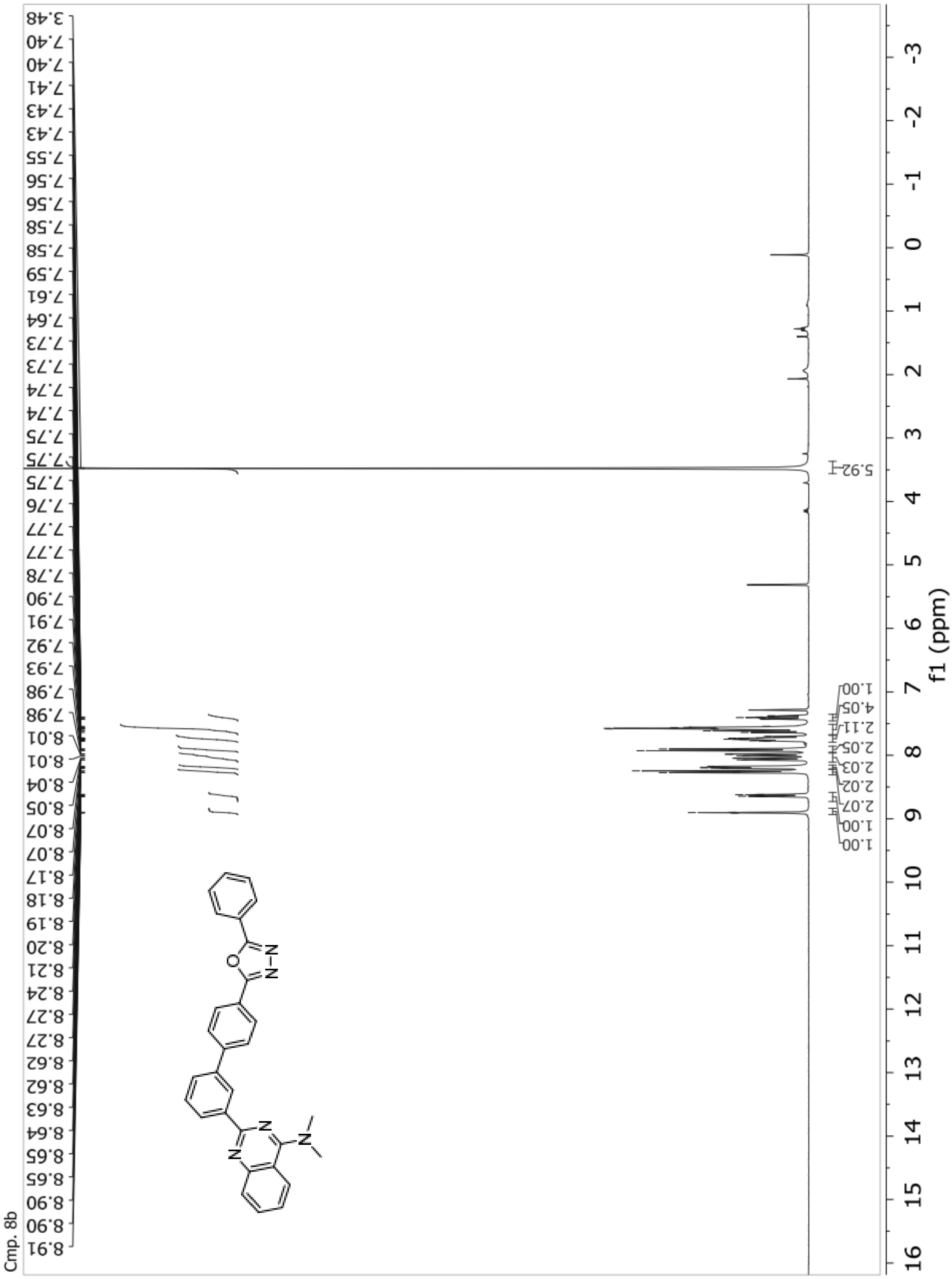

^{13}C NMR spectrum of *N*,*N*-Dimethyl-2-(4'-(5-phenyl-1,3,4-oxadiazol-2-yl)biphenyl-3-yl)quinazolin-4-amine (8b):

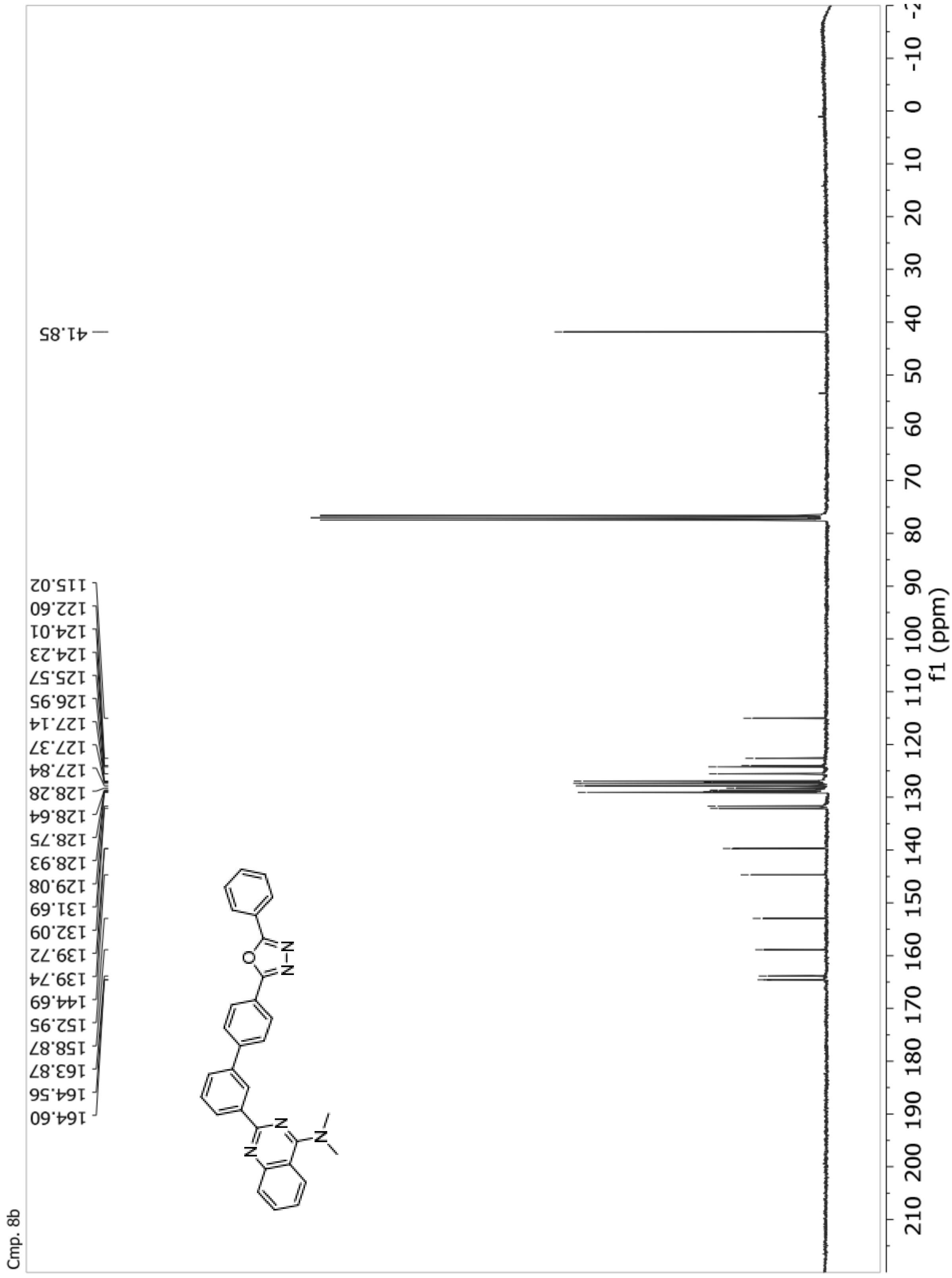

^{1}H NMR spectrum of *N*,*N*-Dimethyl-2-(4'-(5-phenyl-1,3,4-oxadiazol-2-yl)biphenyl-2-yl)quinazolin-4-amine (8c):

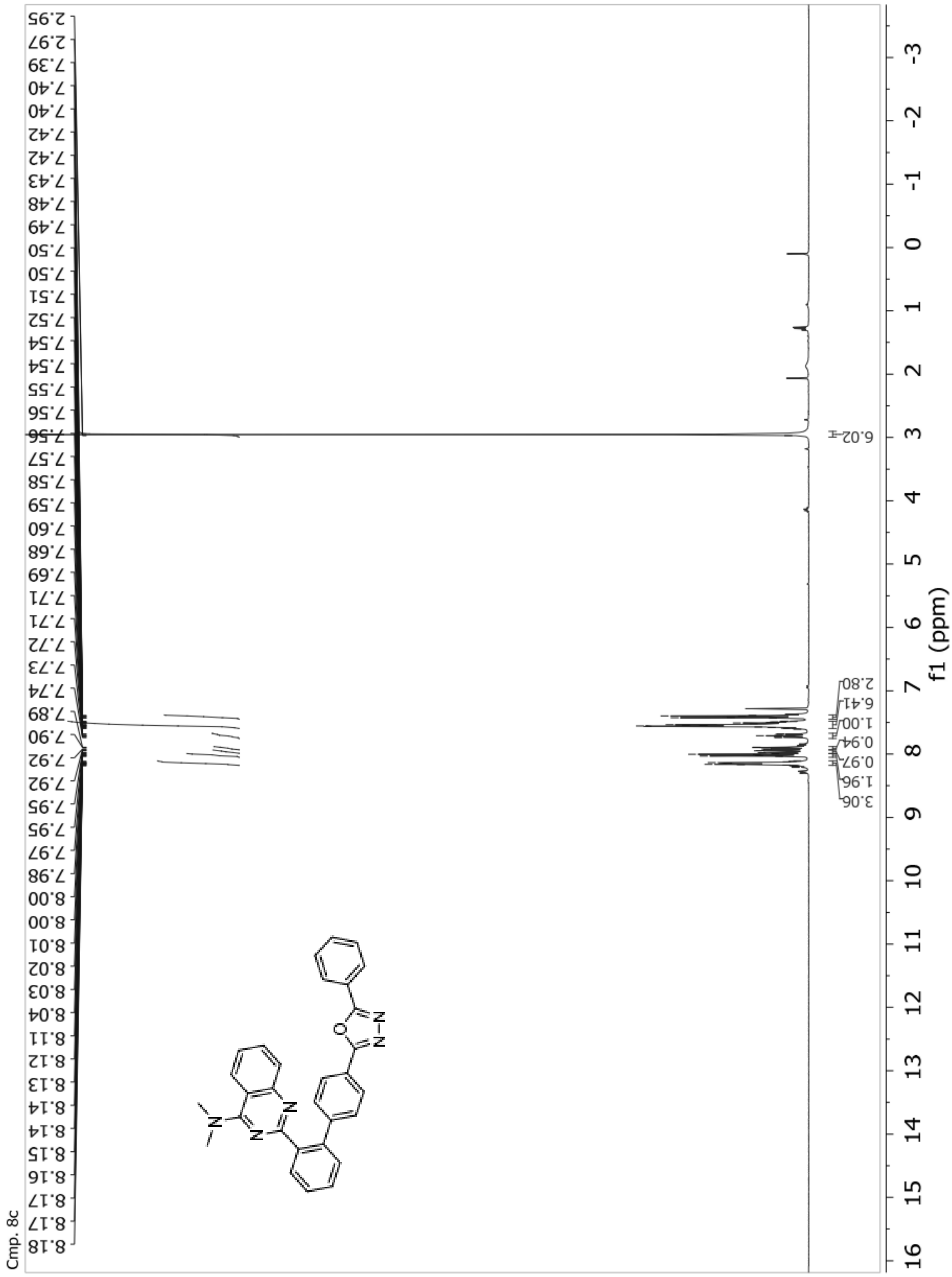

^{13}C NMR spectrum of *N*,*N*-Dimethyl-2-(4'-(5-phenyl-1,3,4-oxadiazol-2-yl)biphenyl-2-yl)quinazolin-4-amine (8c):

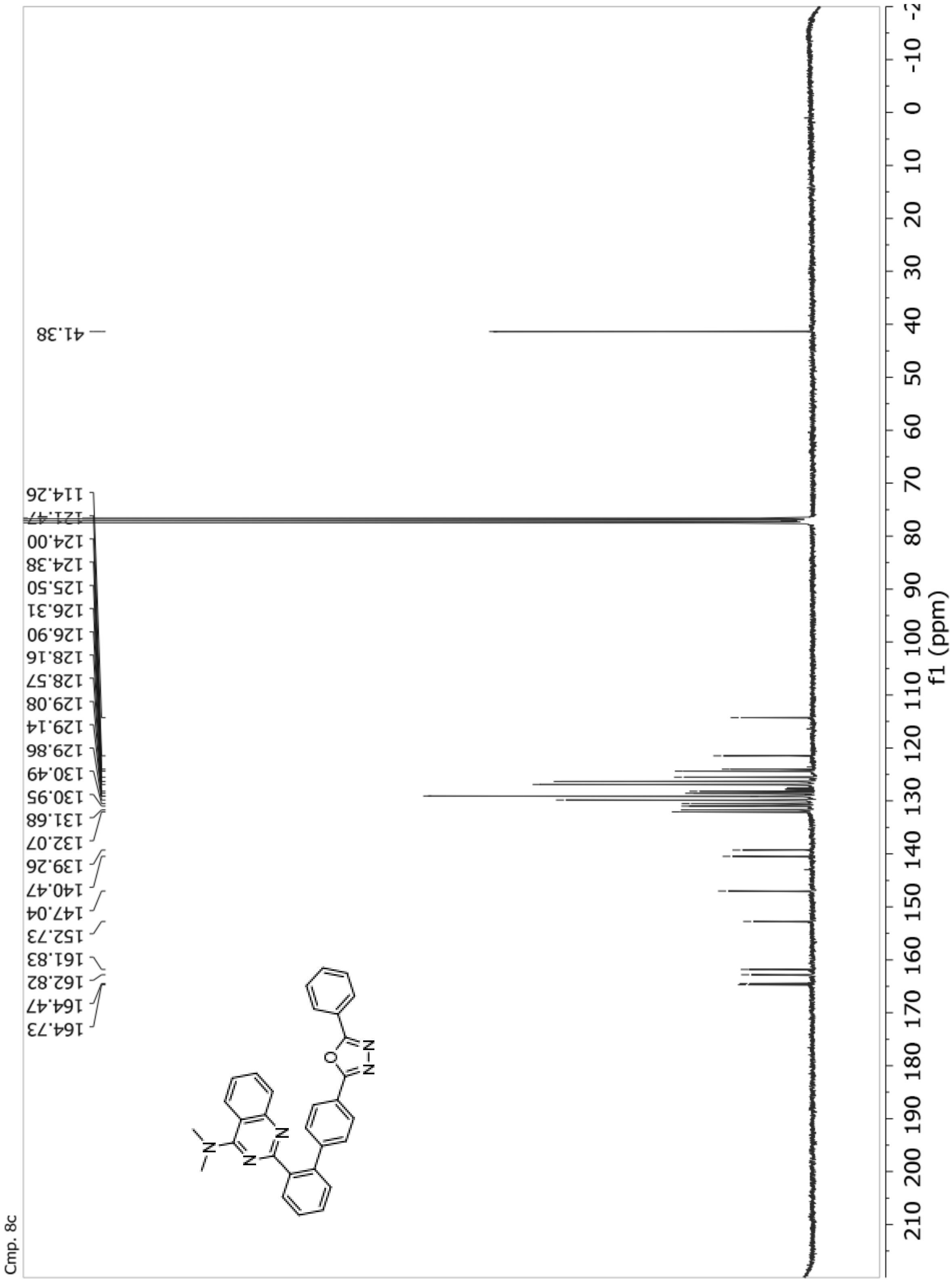

^{1}H NMR spectrum of *N*,*N*-Dimethyl-2-phenyl-6-(4-(5-phenyl-1,3,4-oxadiazol-2-yl)phenyl)quinazolin-4-amine (8f):

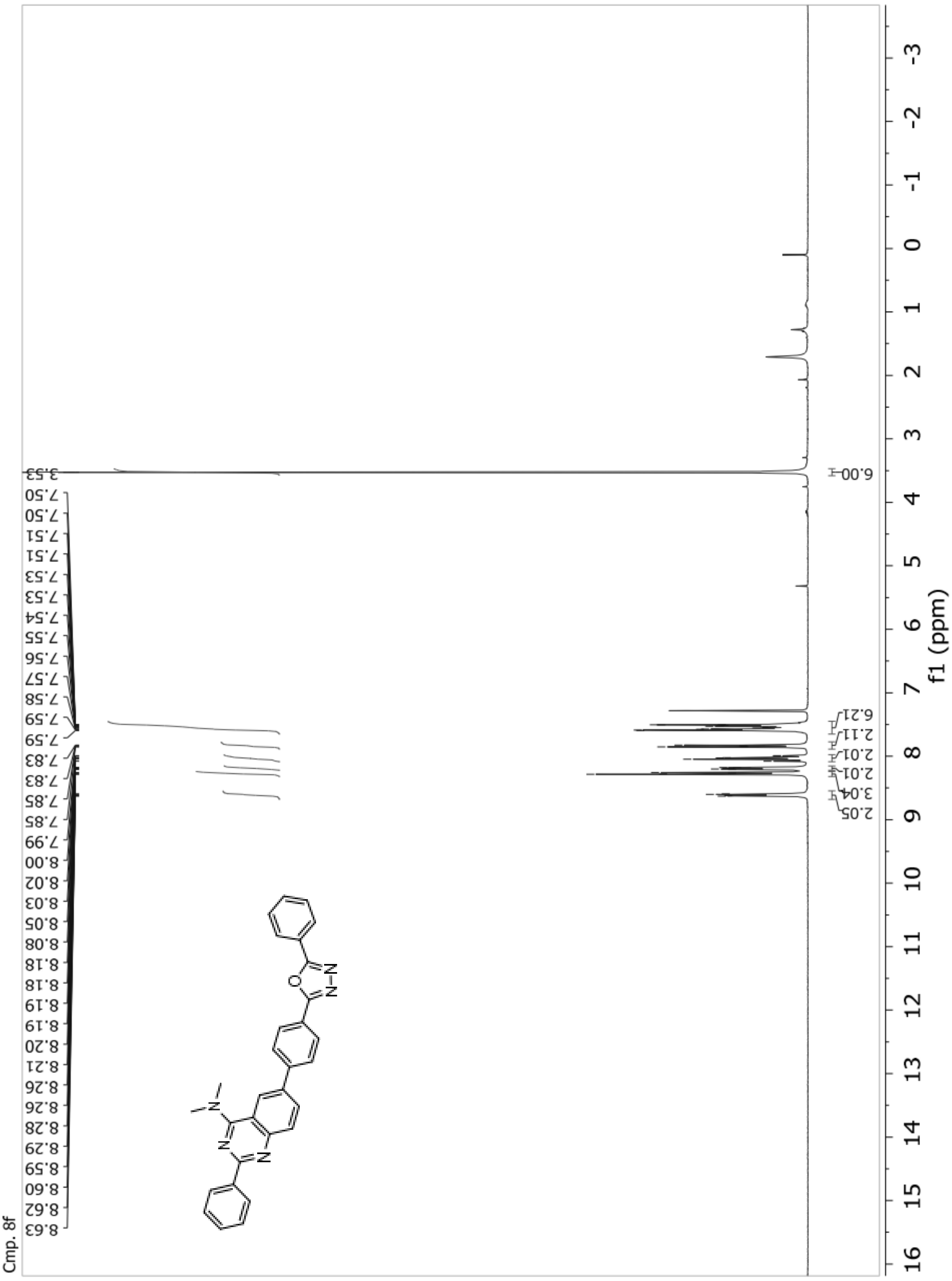

^{13}C NMR spectrum of *N*,*N*-Dimethyl-2-phenyl-6-(4-(5-phenyl-1,3,4-oxadiazol-2-yl)phenyl)quinazolin-4-amine (8f):

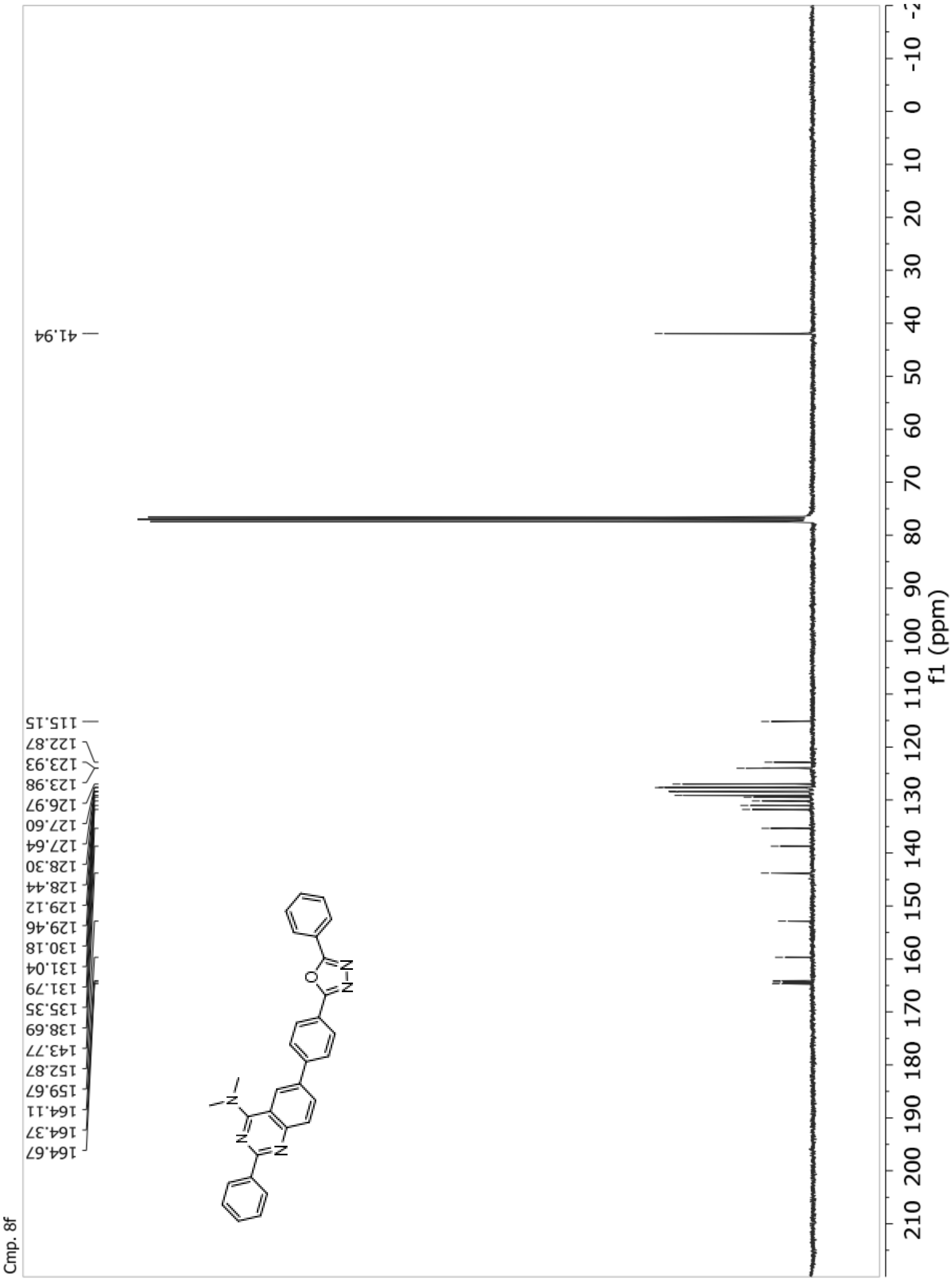

^{1}H NMR spectrum of *N*,*N*-Dimethyl-2-phenyl-7-(4-(5-phenyl-1,3,4-oxadiazol-2-yl)phenyl)quinazolin-4-amine (8g):

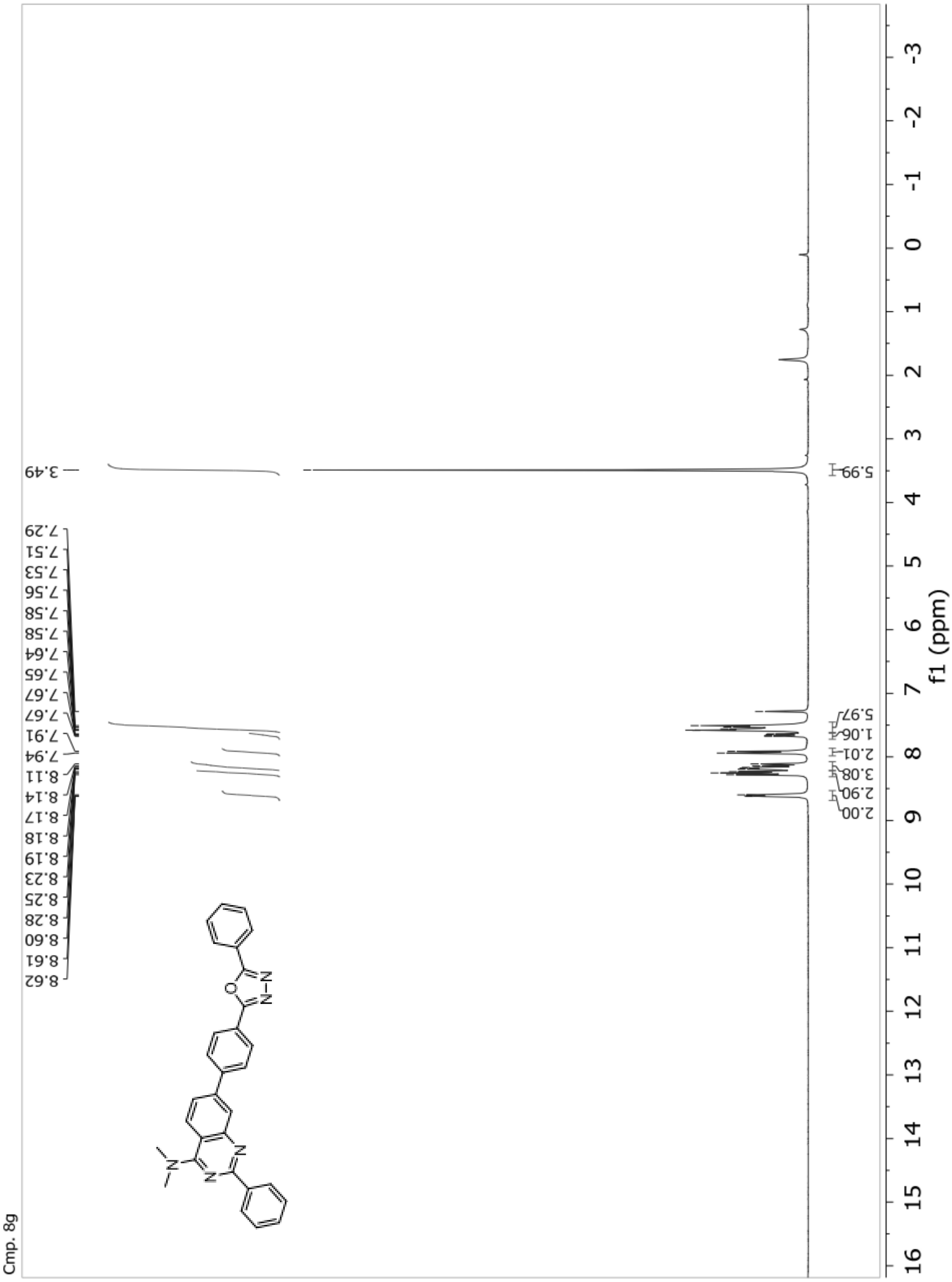

^{13}C NMR spectrum of *N*,*N*-Dimethyl-2-phenyl-7-(4-(5-phenyl-1,3,4-oxadiazol-2-yl)phenyl)quinazolin-4-amine (8g):

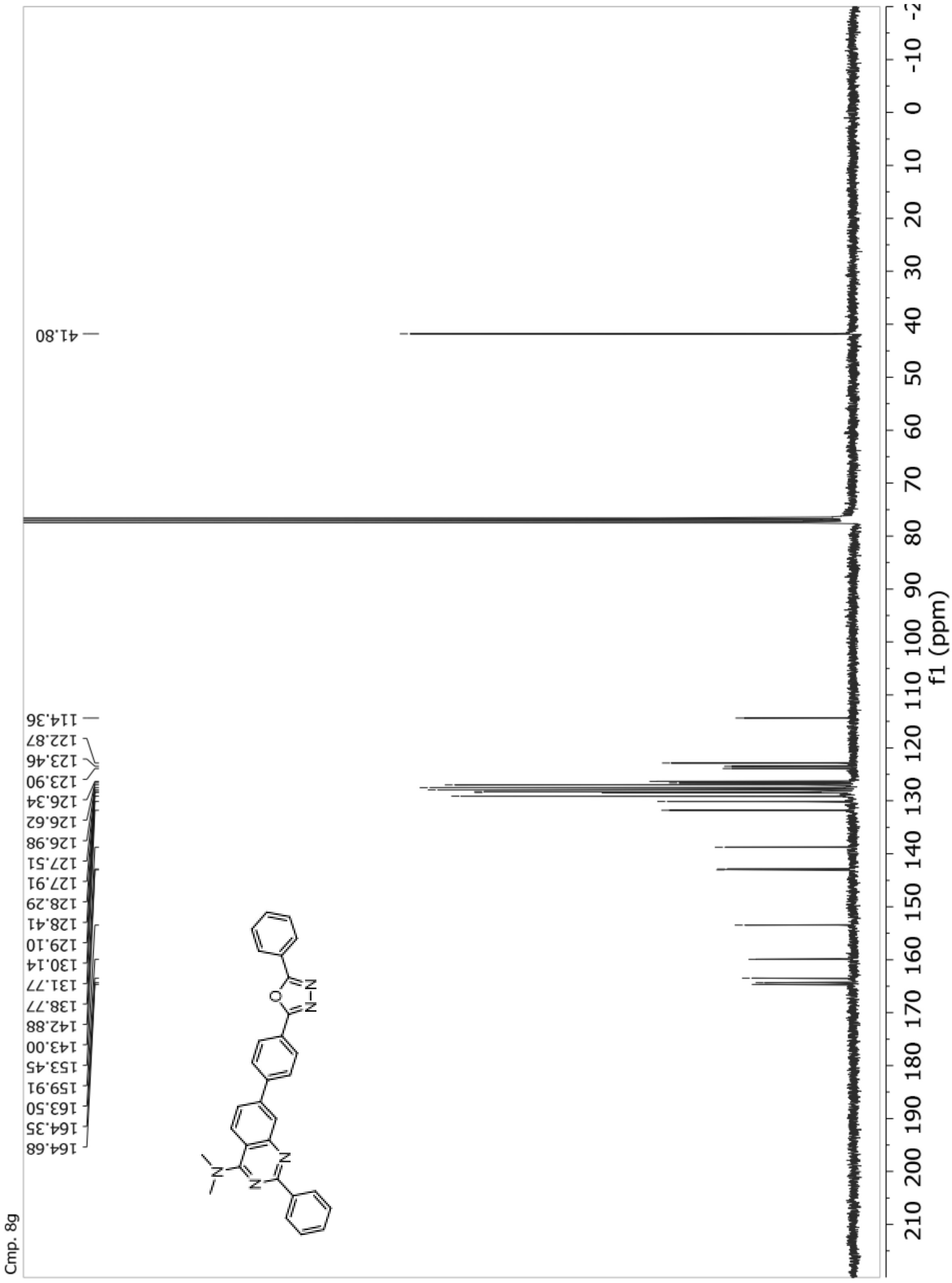

^{1}H NMR spectrum of 2,2'-(4',4''-(1,3,4-Oxadiazole-2,5-diyl)bis(biphenyl-4',4-diyl))bis(*N*,*N*-dimethylquinazolin-4-amine) (9a):

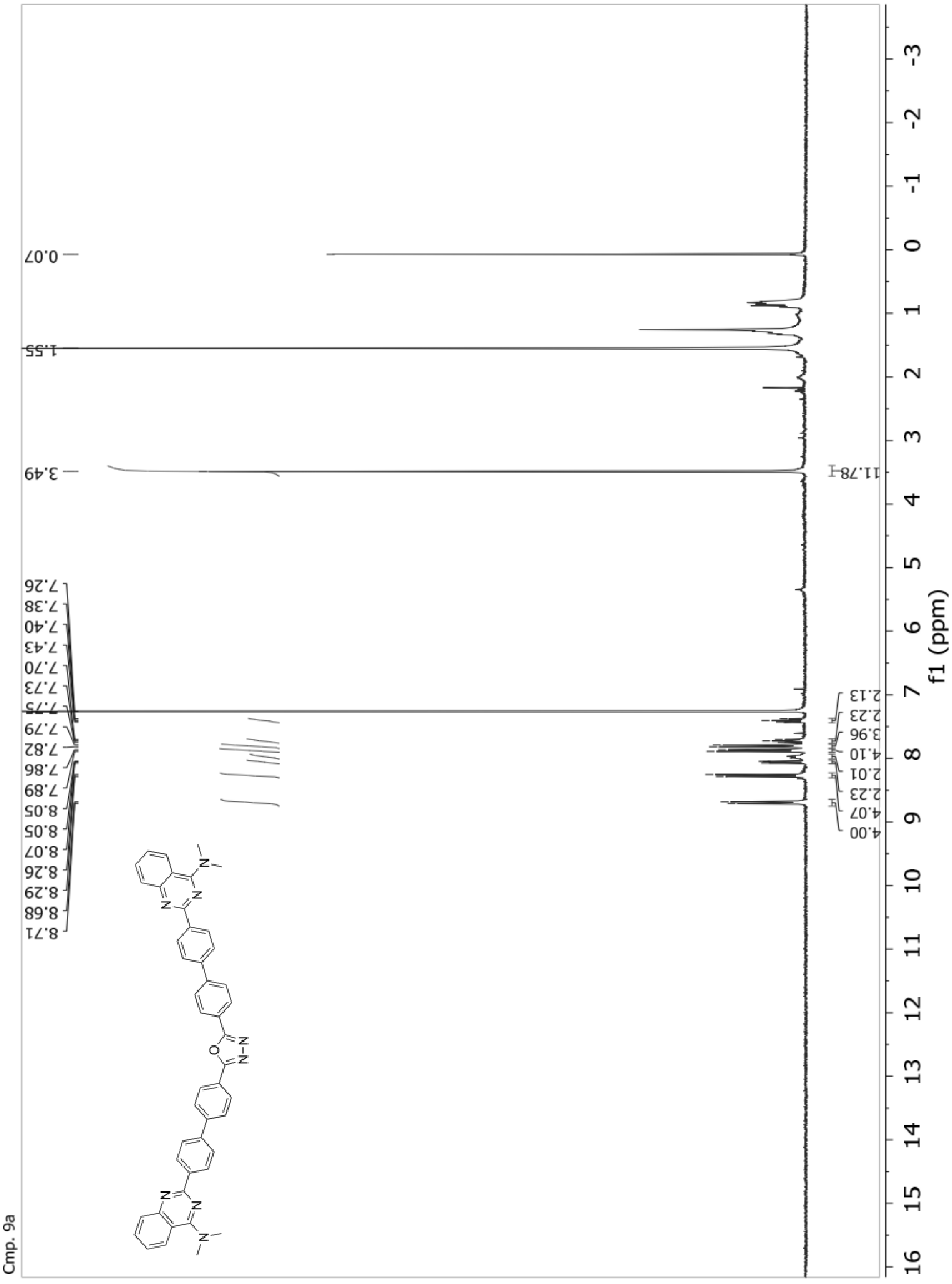

^{13}C NMR spectrum of 2,2'-(4',4''-(1,3,4-Oxadiazole-2,5-diyl)bis(biphenyl-4',4-diyl))bis(*N*,*N*-dimethylquinazolin-4-amine) (9a):

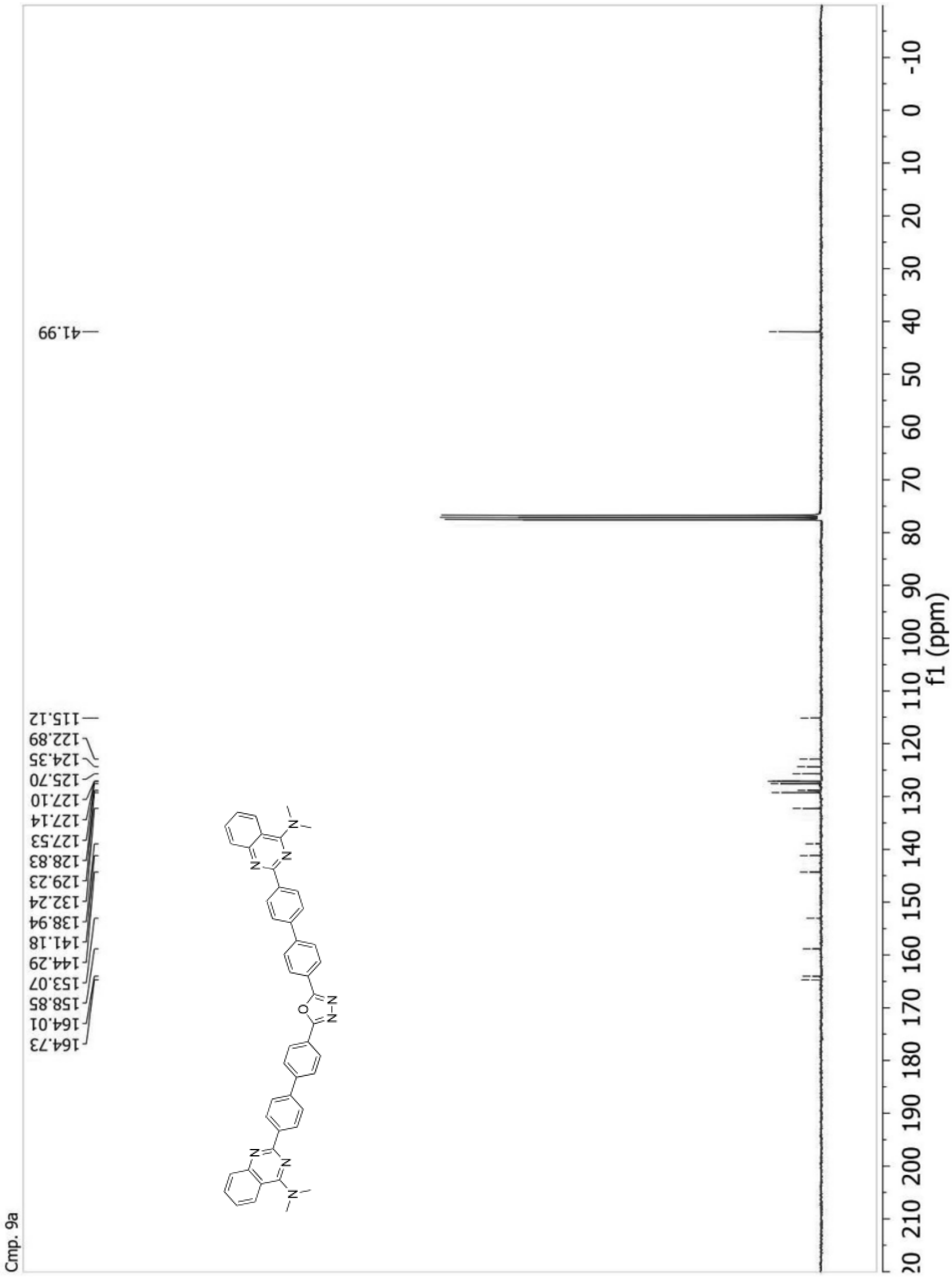

^{1}H NMR spectrum of 2,2'-(4',4''-(1,3,4-Oxadiazole-2,5-diyl)bis(biphenyl-4',3-diyl))bis(*N*,*N*-dimethylquinazolin-4-amine) (9b):

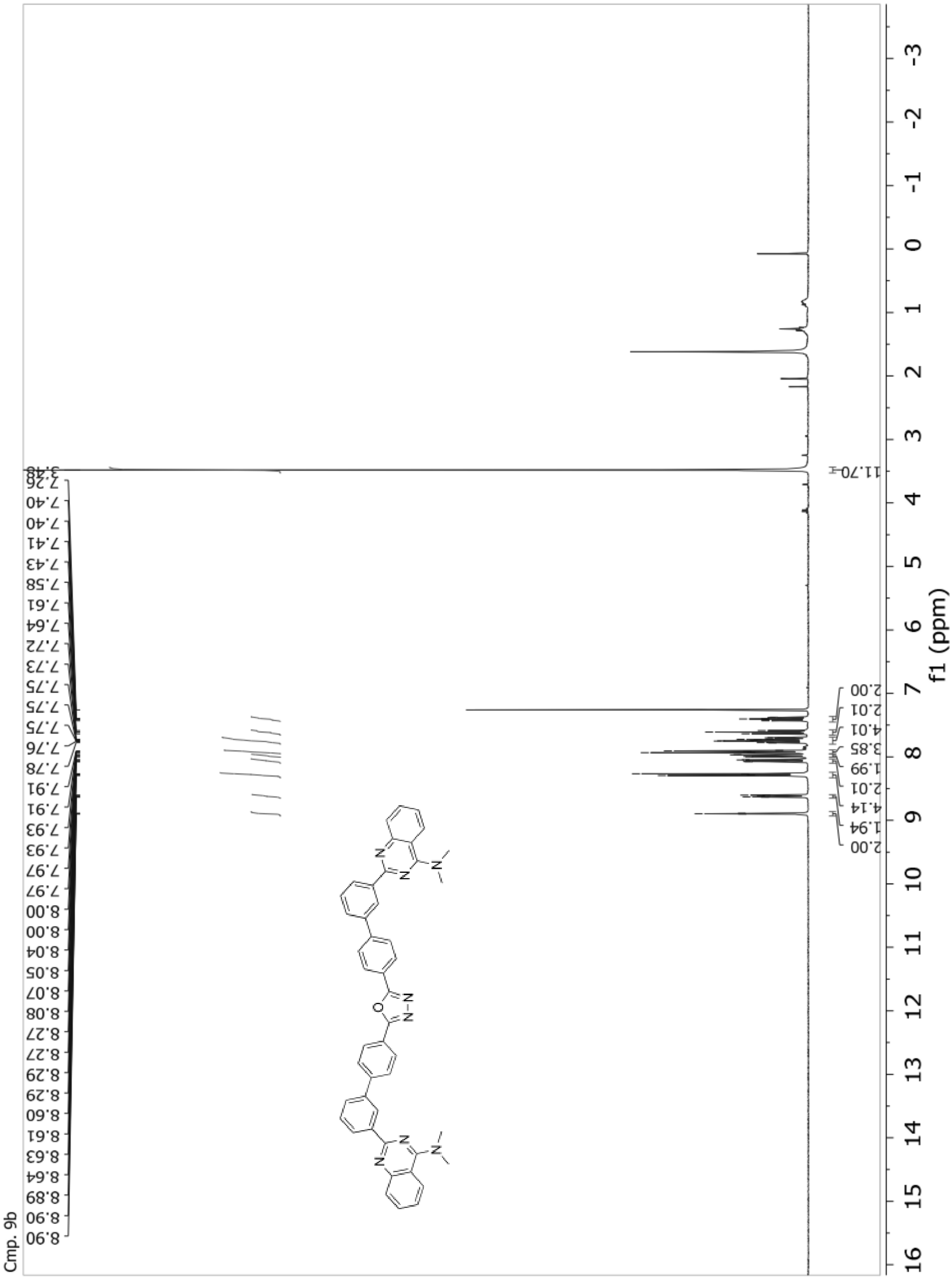

^{13}C NMR spectrum of 2,2'-(4',4''-(1,3,4-Oxadiazole-2,5-diyl)bis(biphenyl-4',3-diyl))bis(*N*,*N*-dimethylquinazolin-4-amine) (9b):

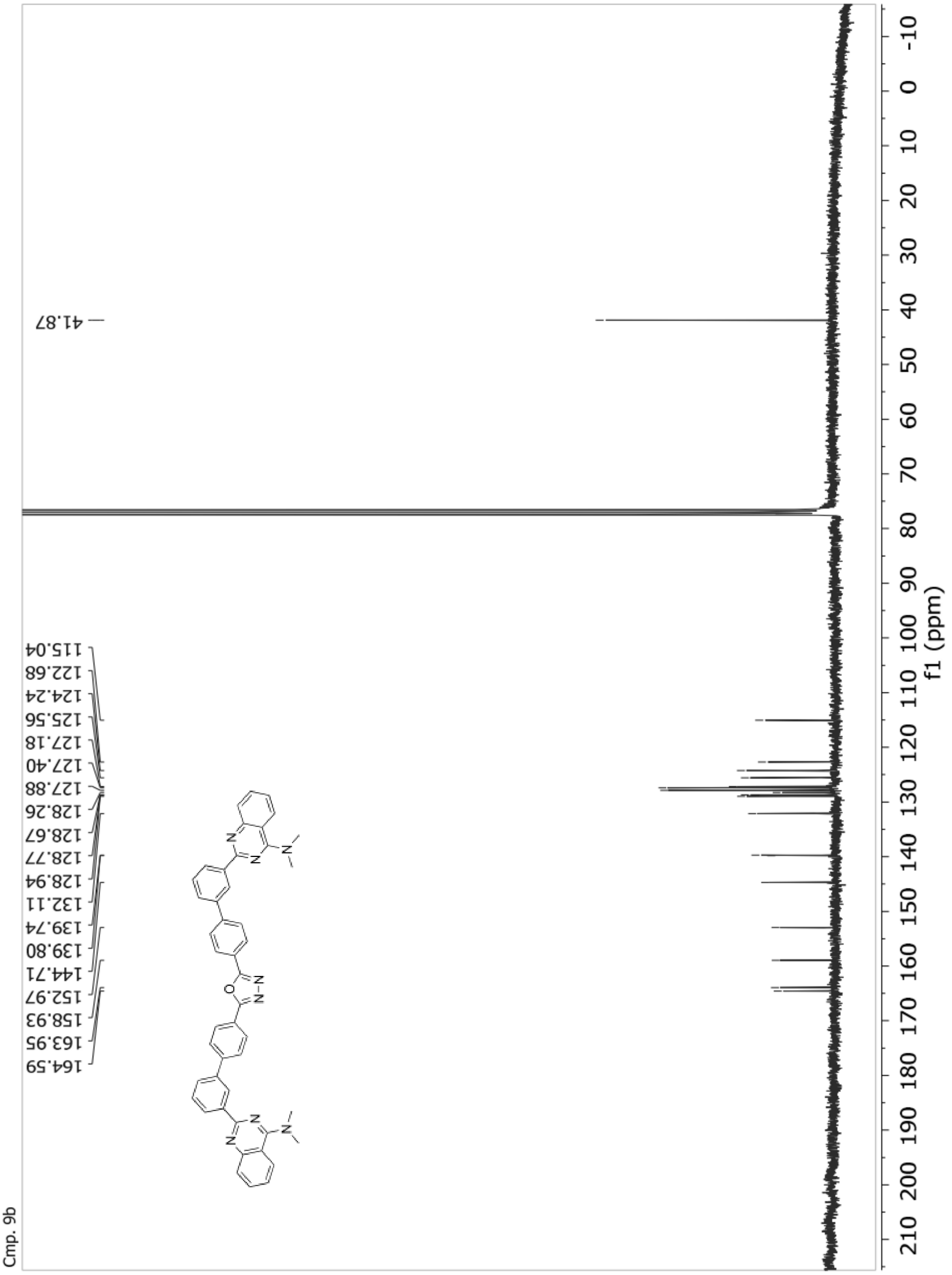

^{1}H NMR spectrum of 2,2'-(4',4''-(1,3,4-Oxadiazole-2,5-diyl)bis(biphenyl-4',2-diyl))bis(*N*,*N*-dimethylquinazolin-4-amine) (9c):

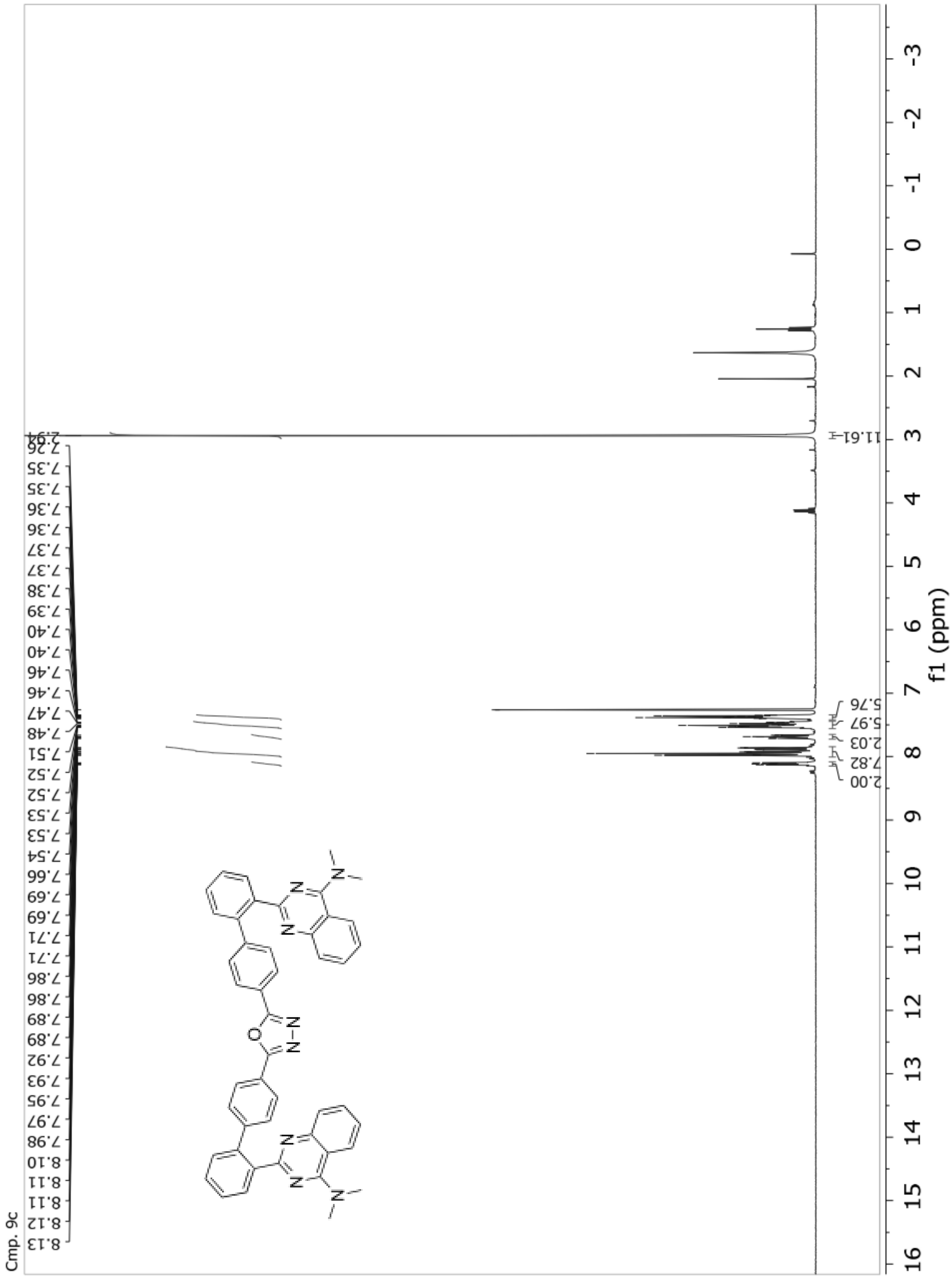

^{13}C NMR spectrum of 2,2'-(4',4''-(1,3,4-Oxadiazole-2,5-diyl)bis(biphenyl-4',2-diyl))bis(*N*,*N*-dimethylquinazolin-4-amine) (9c):

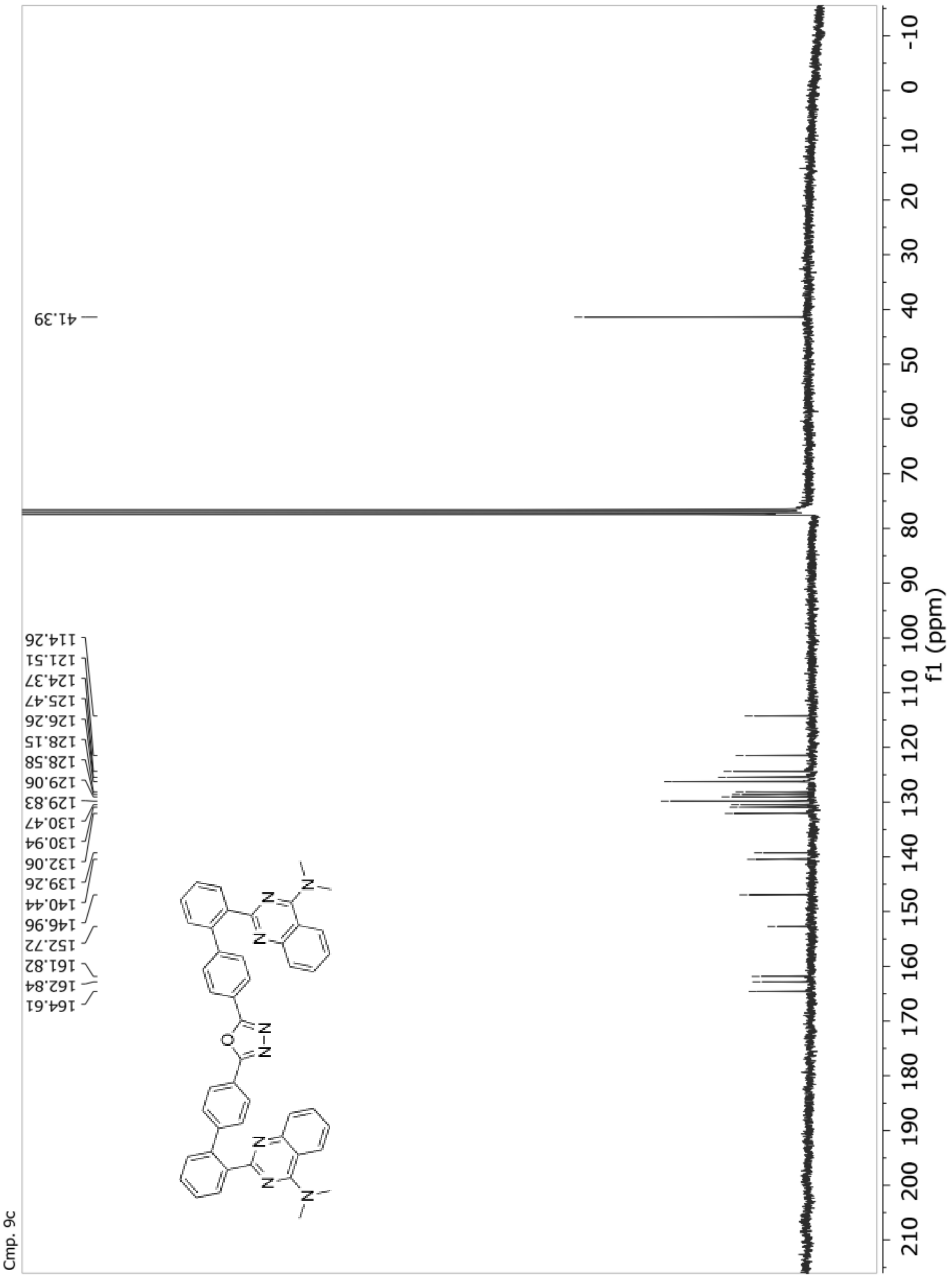

^{1}H NMR spectrum of 6,6'-(4,4'-(1,3,4-Oxadiazole-2,5-diyl)bis(4,1-phenylene))bis(*N*,*N*-dimethylquinazolin-4-amine) (9f):

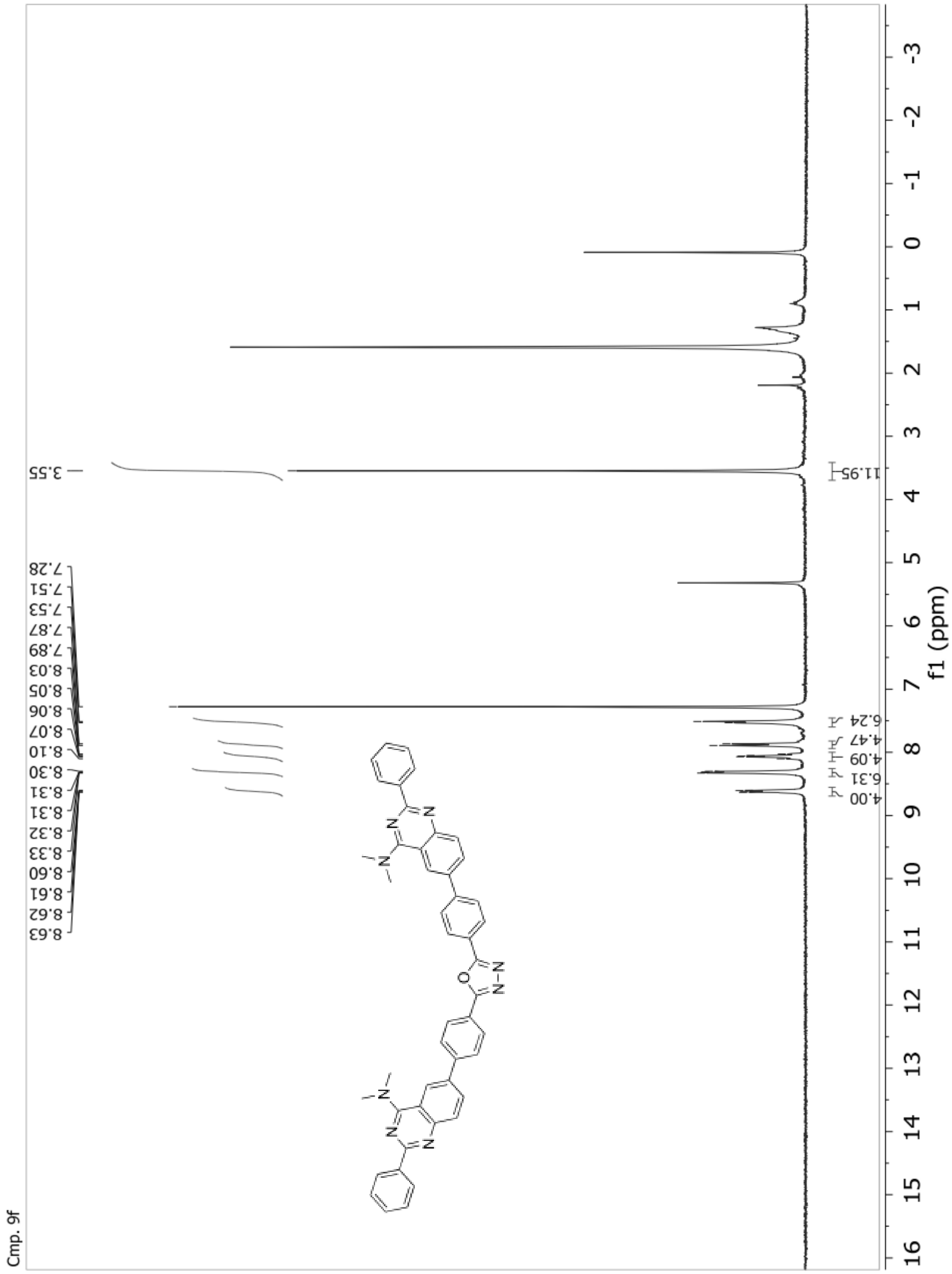

^{13}C NMR spectrum of 6,6'-(4,4'-(1,3,4-Oxadiazole-2,5-diyl)bis(4,1-phenylene))bis(*N*,*N*-dimethylquinazolin-4-amine) (9f):

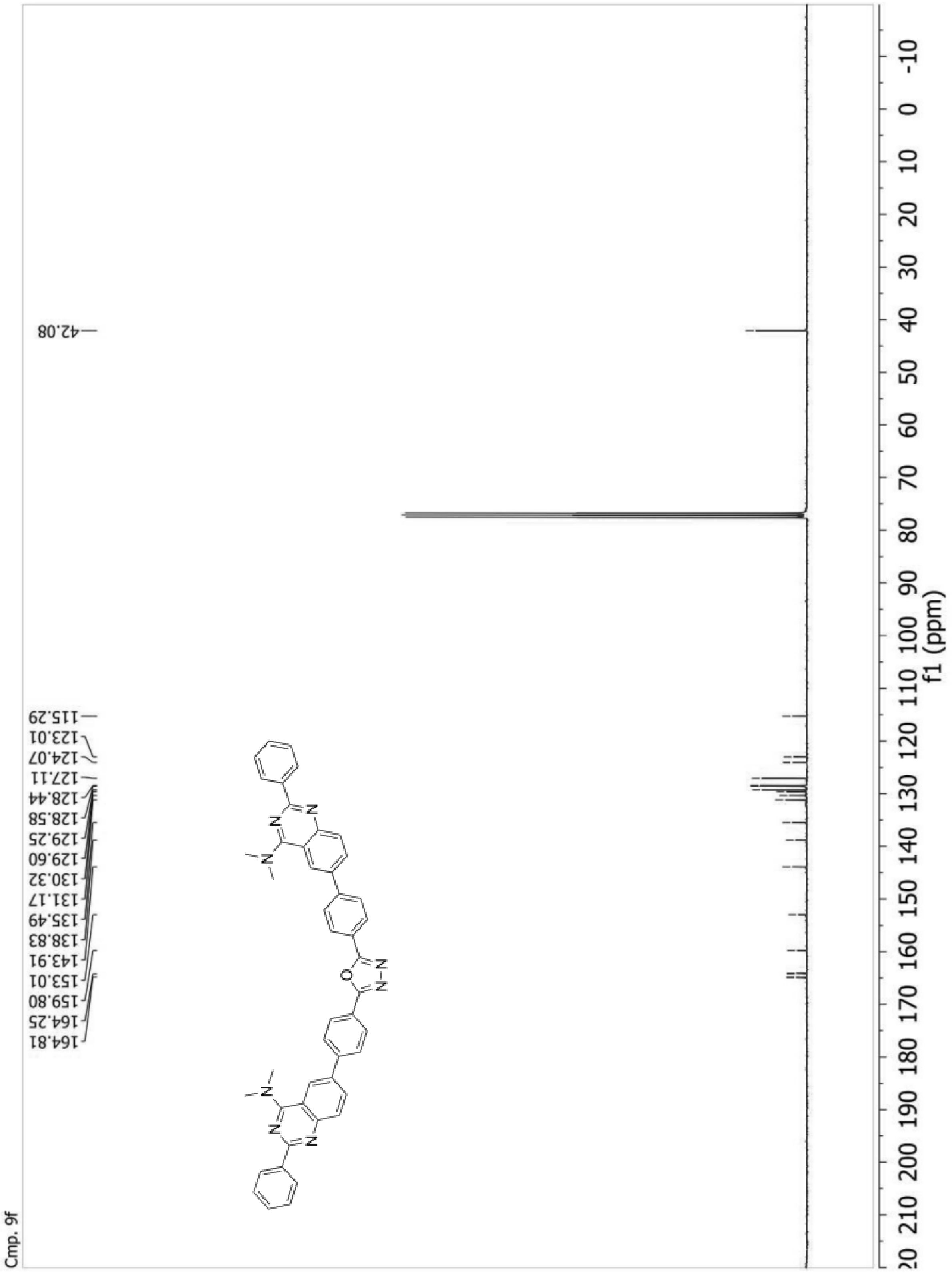

^{1}H NMR spectrum of 7,7'-(4,4'-(1,3,4-Oxadiazole-2,5-diyl)bis(4,1-phenylene))bis(*N*,*N*-dimethylquinazolin-4-amine) (9g):

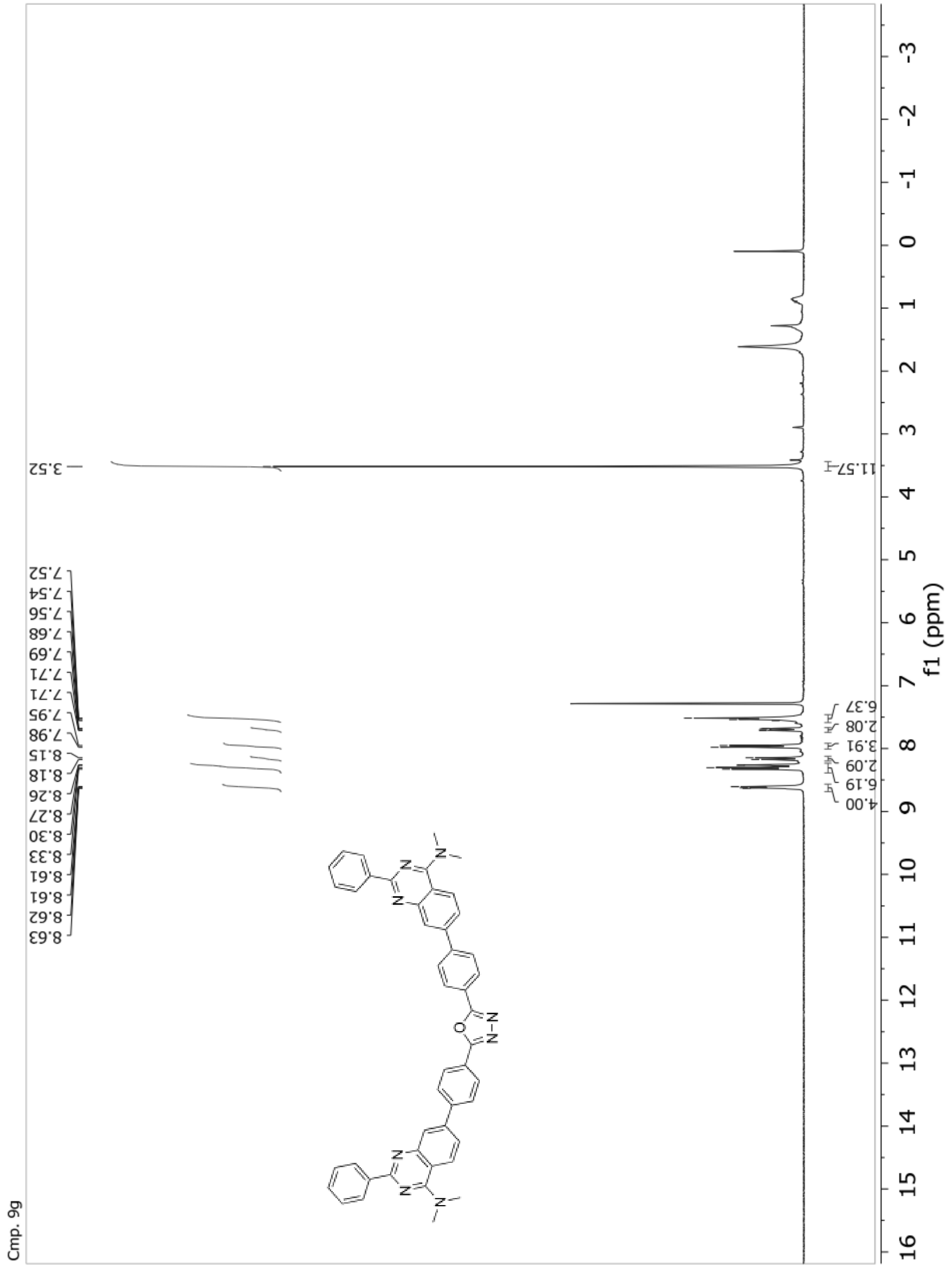

^{13}C NMR spectrum of 7,7'-(4,4'-(1,3,4-Oxadiazole-2,5-diyl)bis(4,1-phenylene))bis(*N*,*N*-dimethylquinazolin-4-amine) (9g):

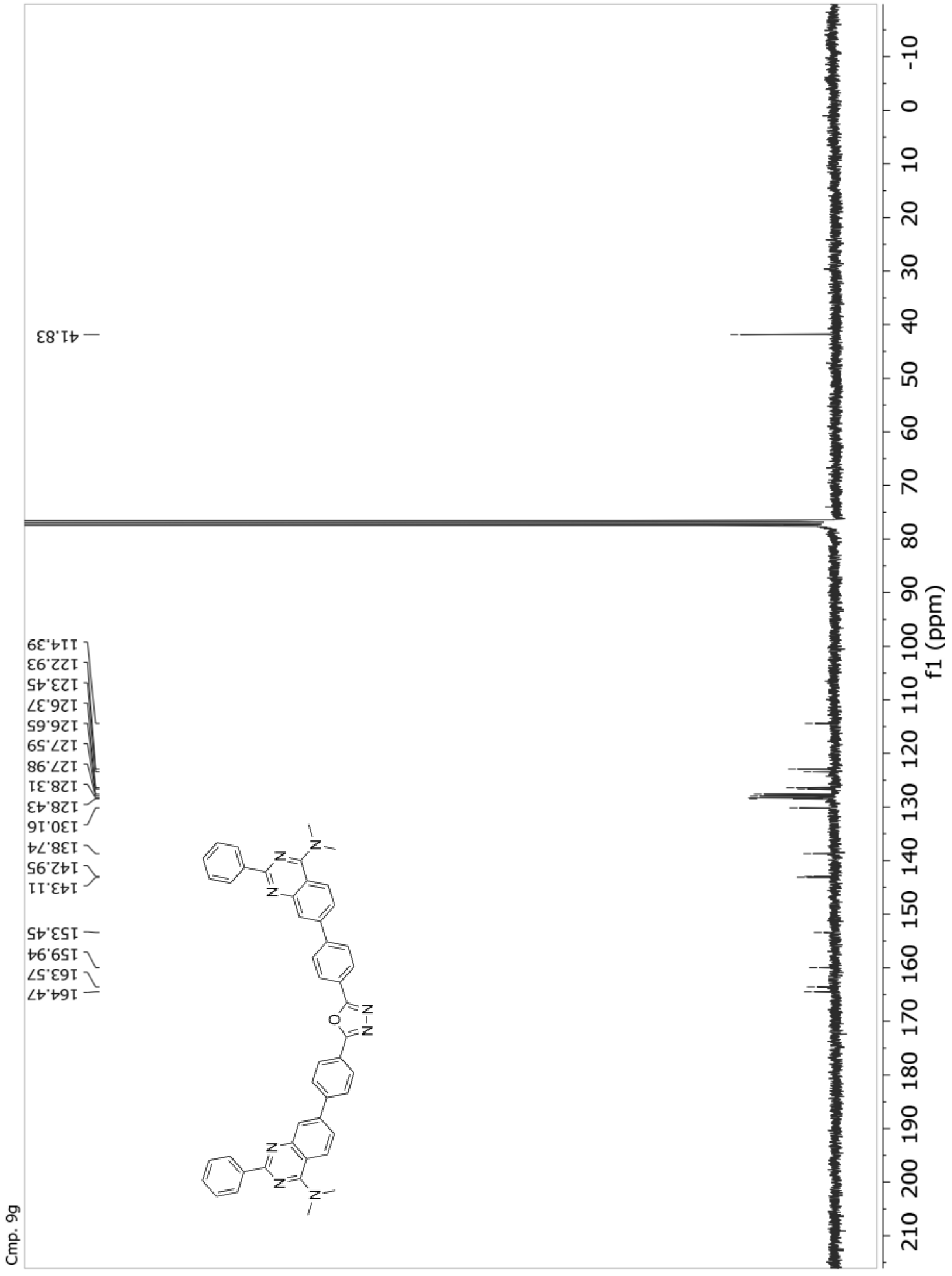

Supplement: Supplementary file 1 [file molecules-25-05150-s001.pdf]
